# Efficacy and safety of Shexiang Baoxin Pill for stable coronary artery disease: A systematic review and meta-analysis of 42 randomized controlled trials

**DOI:** 10.3389/fphar.2022.1002713

**Published:** 2022-11-14

**Authors:** Jingjing Wei, Teng Ma, Cheng Zhou, Pengle Hao, Bin Li, Xinlu Wang, Rui Yu, Mingjun Zhu, Yongxia Wang

**Affiliations:** ^1^ The First Affiliated Hospital of Henan University of Chinese Medicine, Zhengzhou, Henan, China; ^2^ Department of Cardiovascular Diseases, The First Affiliated Hospital of Henan University of Chinese Medicine, Zhengzhou, Henan, China; ^3^ Second Teaching Hospital of Tianjin University of Traditional Chinese Medicine, Tianjin, China

**Keywords:** Shexiang Baoxin Pill, stable coronary artery disease, randomized controlled trials, meta-analysis, grade

## Abstract

**Objective:** Patients with stable coronary artery disease (SCAD) still have a higher risk of adverse cardiovascular events. Shexiang Baoxin Pill (SBP) is widely used as a complementary and alternative treatment for SCAD. This study aimed to further verify the therapeutic effect and safety of SBP on SCAD.

**Methods:** Seven databases were involved in this meta-analysis as of 1 June 2022. Data was collected from all the randomized controlled trials (RCTs) of the combination of SBP and conventional western medicine (CWM) in treating SCAD which was conducted by two independent authors. Risk of bias was assessed using the Cochrane risk-of-bias 2.0 (RoB2.0) tool, and the meta-analysis was accomplished with Review Manager 5.3. Furthermore, the Grading of Recommendations Assessment, Development and Evaluation (GRADE) profiler 3.2.2 software was selected to grade the current evidence in our findings.

**Results:** 42 articles, involving 6,694 patients were screened among all the 1,374 records in the analysis. The results demonstrated that the combination therapy was more efficient than CWM alone in lowering the incidence of major adverse cardiovascular events (MACE, RR = 0.50, 95% CI: 0.37 to 0.68, *p* < 0.00001) and ameliorating the total effective rate of angina symptom improvement (RR = 1.23, 95% CI: 1.19 to 1.28, *p* < 0.00001), the effective rate of electrocardiogram improvement (RR = 1.34, 95% CI: 1.26 to 1.43, *p* < 0.00001), the frequency of angina pectoris (MD = −2.83, 95% CI: −3.62 to −2.05, *p* < 0.00001), and the duration of angina pectoris (MD = −1.32, 95% CI: −2.04 to −0.61, *p* = 0.0003). We also found that, after SBP treatment, a more positive blood lipid level and left ventricular ejection fraction without the increase in adverse cases were calculated in our meta-analysis. What’s more, Subgroup analysis indicated that treatment duration may be the source of heterogeneity. The certainty of the evidence for MACE, and electrocardiogram improvement exhibited moderate certainty, and the certainty of the evidence for the remaining outcomes was judged as low certainty. The trial sequential analysis further affirmed the clinical efficacy of SBP.

**Conclusion:** The available evidence indicates that SBP may be an effective therapeutic option in patients with SCAD. However, considering the inferior quality and inconsistent results in the included trials, further rigorous RCTs are required.

**Systematic Review Registration:** https://www.crd.york.ac.uk/prospero, identifier [CRD42022334529].

## 1 Introduction

Stable coronary artery disease (SCAD) is the most common type of coronary heart disease (CHD), mainly including stable angina pectoris, stable phase after acute coronary syndrome, and ischemic cardiomyopathy (Montalescot et al., 2013). A report from the American Heart Association (AHA) in 2016 showed that the incidence of SCAD is much higher than that of myocardial infarction, which is twice as high as that of myocardial infarction and is expected to reach 18% of the adult population by 2030 (Mozaffarian et al., 2016). Notably, SCAD can remain stable for a long time or can become unstable at any time due to plaque rupture or erosion leading to acute coronary events. Currently, aspirin and statins are the standard secondary prevention approach in reducing the risk of cardiovascular events in patients with SCAD. However, patients who receive secondary prevention still have a 4%–12% risk of major adverse cardiovascular events (MACE), and there is still a considerable residual cardiovascular risk (Bhatt et al., 2010; Feres et al., 2013). How to further reduce the risk of recurrent cardiovascular events in SCAD remains a hot spot and a challenge for current research.

In recent years, with the increasing clinical evidence of traditional Chinese medicine (TCM) for the treatment of CHD, TCM may become a supplementary and alternative medicine for the primary and secondary prevention of patients with SCAD (Liang and Gu, 2021). Shexiang Baoxin Pill (SBP) is currently one of the most commonly used aromatic medicines for the treatment of cardiovascular diseases in China, which has been widely used to relieve and prevent angina-related symptoms since its marketing in 1981 (Guo et al., 2021; Wei et al., 2021). SBP is a Chinese medicine compound prescription composed of Moschus (the dried preputial secretion of *Moschus berezovskii, M. sifanicus or M. moschiferus*), Radix Ginseng (*Panax ginseng C.A.Mey.*), Bovis Calculus Artifactus (the dried gall-stone of *Bos taurus domesticus Gmelin*), Cinnamomi Cortex (*Cinnamomum cassia*), Styrax (*Liquidambar orientalis Mill.*), Bufonis Venenum (*Bufo gargarizans*), and Borneolum Syntheticum (*Dryobalanops aromatica C.F.Gaertn.*) (Supplementary Material S1). It has been recommended for the treatment of CHD by the “Guidelines for TCM diagnosis and treatment of stable angina pectoris of coronary heart disease” (China Association of Chinese Medicine Cardiovascular Disease Branch, 2019). Network pharmacology analysis found that SBP and its plasma absorption compounds can dilate blood vessels by upregulating cyclooxygenase-2 and downregulating intercellular adhesion molecule-1 (Fang et al., 2017). Pharmacological studies have shown that SBP has a protective effect on damaged vascular endothelial cells, and can inhibit inflammation of the vascular wall and stabilize atherosclerotic plaques during the atherosclerotic process (Lu et al., 2019; Zhang et al., 2020). Recent studies have found that SBP can affect the endothelial cell signaling pathway to promote the expression of therapeutic angiogenesis-related genes, and it is speculated that this mechanism may be related to the compounds such as ginsenosides and cinnamaldehyde contained in it (Hu et al., 2021). A previous review evaluated the relevant randomized Controlled Trials (RCTs) before December 2017 and showed the efficacy and safety of SBP in the treatment of stable angina (Pan et al., 2019). A subsequent review reported that SBP combined with conventional therapy can improve coronary microvascular function (Wang et al., 2021). In recent years, several research trials have focused on the clinical efficacy and safety of SBP as an additional treatment for SCAD (Wang, 2016; Peng et al., 2017). In particular, a multicenter, double-blind, placebo-controlled phase IV randomized clinical trial in 2021 reaffirmed the clinical value of SBP in patients with SCAD (Ge et al., 2020). Regrettably, there are no relevant systematic reviews to summarize the efficacy and safety of SBP in the treatment of SCAD in terms of both methodological quality and quality of evidence.

Therefore, we conducted a systematic review and meta-analysis based on the available evidence to strictly evaluate the efficacy and safety of SBP for SCAD, and to clarify the strength of the evidence for SBP, to better guide clinical application.

## 2 Methods

### 2.1 Program and registration

This report adhered to the Preferred Reporting Items for Systematic Reviews and Meta-Analyses 2020 statement (Page et al., 2021) (Supplementary Material S2). We have already registered our protocol on the PROSPERO (number: CRD42022334529).

### 2.2 Search strategy

Two investigators independently searched the databases including PubMed, The Cochrane Library, Web of Science, China National Knowledge Infrastructure (CNKI), China Science and Technology Journal Database (VIP), WanFang Database, and SinoMed from inception until 1 June 2022. The method for searching was based on subject headings combined with free words. In addition, we also manually retrieved the reference lists of published literature to search for other relevant studies. Unpublished literature was identified by searching the websites of national and international medical specialty societies, clinical trial registration platforms, and clinical practice guideline collections. The search strategies were formulated by physicians (MZ and YW), and statisticians (BL and XW). We have provided detailed search strategies in Supplementary Material S3.

### 2.3 Inclusion and exclusion criteria

#### 2.3.1 Types of research

Only RCTs of SBP for patients with SCAD were included and not restricted by language or publication type.

#### 2.3.2 Types of participants

All subjects (age ≥18 years) of the included study meet the diagnostic criteria for SCAD established by the AHA, the European Society of Cardiology, or the Chinese Medical Association (Fihn et al., 2012; Montalescot et al., 2013; Interventional Cardiology Group of Cardiovascular Branch of Chinese Medical Association., 2018). No restrictions were made regarding gender, country, or race.

#### 2.3.3 Types of interventions

The experimental group and control group were both treated with antiplatelet drugs, lipid-lowering drugs, vasodilators of nitrate, and other conventional western medicine (CWM) recommended by the guideline (Interventional Cardiology Group of Cardiovascular Branch of Chinese Medical Association., 2018). The experimental group was added with SBP (produced by Shanghai Hutchison Pharmaceuticals Co., Ltd.) for adjuvant therapy.

#### 2.3.4 Types of outcomes

The primary outcomes are as follows: 1) MACE including cardiovascular death, nonfatal myocardial infarction, and nonfatal stroke; 2) the total effective rate of angina symptom improvement (significant effectiveness: symptom basically disappeared, and the number of angina attacks decreased by at least 80%; effective: symptom improved significantly, and the number of angina attacks decreased by 50%–80%; inefficacy: no significant improvement in symptom, less than 50% reduction in the number of angina attacks) (according to “Guiding Principles for Clinical Research of New Chinese Medicines”) (Ministry of health of the people’s republic of China, 2002); 3) electrocardiogram (ECG) improvement (significant effectiveness: ECG returned to the normal range; effective: ST segment was reduced, and after treatment, it rose above 0.05 mV but did not reach the normal level, or the T wave changed from a flat state to an upright state; inefficacy: the ECG had no significant changes compared to before treatment; worsening of disease: the ST segment was reduced by more than 0.05 mV after treatment; the T wave state was completely changed, and ectopic heart rate occurred); 4) adverse events (AEs) including clinical symptoms, signs, laboratory abnormalities.

The secondary outcomes are as follows: 1) angina pectoris frequency: in the unit of times/week; 2) angina pectoris duration: in the unit of min/time; 3) left ventricular ejection fraction (LVEF); 4) blood lipid level including total cholesterol (TC), triglyceride (TG), low-density lipoprotein cholesterol (LDL-C), and high-density lipoprotein cholesterol (HDL-C).

#### 2.3.5 Exclusion criteria

The exclusion criteria were as follows: 1) duplicate published literature; 2) the included studies did not report the outcomes of interest of this systematic review; 3) the baseline information of patients was inconsistent; 4) intervention measures combined with other TCM.

### 2.4 Literature screening and data extraction

The literature retrieved from each database is imported into Endnote software for deduplication, and two researchers screen the literature according to the inclusion and exclusion criteria, extract the information, and recheck each other’s work. If there is any disagreement, the third researcher will be invited to discuss and make a decision. Data were extracted using a standardized data extraction form to extract information including first author, year of publication, sample size, gender, age, disease condition, intervention measures, treatment duration, and outcome indicators.

### 2.5 Risk of bias assessment

Two reviewers independently assessed the risk of bias in the RCTs using the Cochrane risk-of-bias 2.0 (RoB2.0) tool (Liu et al., 2019). The assessment of ROB includes the following five domains: 1) bias arising from the randomization process; 2) bias due to deviations from intended interventions; 3) bias due to missing outcome data; 4) bias in the measurement of the outcome; 5) bias in the selection of the reported result. Finally, a judgment of the overall risk of bias is generated. The ROB was judged as “low”, “high”, or “some concerns”. Disagreements between the two researchers were resolved through consultation with a third researcher.

### 2.6 Data analysis

The software Review Manager 5.3 is used for statistical analysis. Cochran’s *Q* and *I* ^2^ are chosen to test for heterogeneity, and if there is statistical heterogeneity among the findings (*I* ^2^ ≥ 50%, *p* < 0.10), a random-effects model will be selected, and conversely, a fixed-effects model will be used. Binary variables were analyzed using relative risk (RR) as the pooled statistic, and continuous variables were analyzed using weighted mean difference (MD) as the pooled statistic, both of which describe the 95% confidence interval (CI). Meta-regression will be performed to find the reasons for heterogeneity, such as publication year, age, course of treatment, and sample size. Further, the statistically significant factors obtained by meta-regression will be used as grouping indicators for subgroup analysis. Sensitivity analyses were performed by omitting each study at a time to assess the consistency and stability of the pooled results. In addition, we examined potential publication bias using Egger’s test method. Finally, the TSA 0.9.5.10 Beta software was used to perform TSA analysis on the associated results.

### 2.7 Assessing the certainty of evidence

The Grading Recommendations Assessment, Development, and Evaluation (GRADE) technique was used to assess the certainty of the evidence (Goldet and Howick, 2013) following the instructions of the website (https://training.cochrane.org/handbook/current/chapter-14/). RCT evidence is initially classified as high quality, but it can be downgraded due to the risk of bias, inconsistency, indirectness, imprecision, and publication bias. The level of evidence is classified into four categories: “high,” “moderate,” “low,” and “very low”.

## 3 Results

### 3.1 Literature search results

**FIGURE 1:**
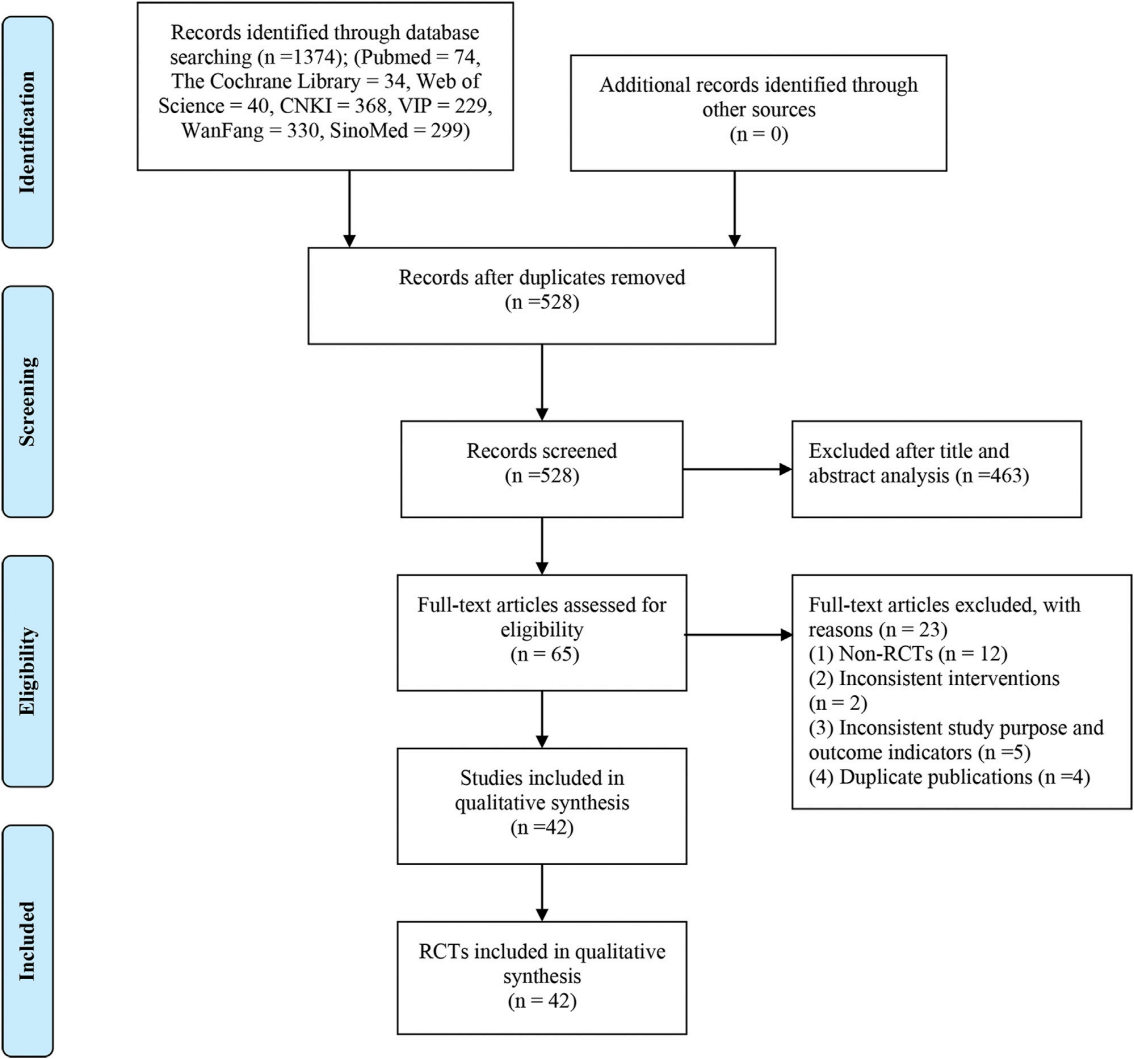
Flow diagram of study selection and identification.

The flowchart of search results is shown in Figure 1 according to the PRISMA study. We included 1,374 records from seven electronic databases. After duplicate studies were removed, we screened 528 titles and abstracts and finally obtained 65 full-text articles. Among them, 23 studies were excluded, of which 12 studies were not RCTs, two studies were inconsistent interventions, five studies did not match the research purpose and outcomes, and four studies were published repeatedly. Supplementary Material S4 contains a list of studies that were excluded by reading the full text. Eventually, 42 eligible studies are included (Sun, 2010; Xu et al., 2010; Shi and Hang, 2011; Guo and Tan, 2012; Jiang, 2012; Wu et al., 2012; Sun, 2013; Yang et al., 2013; Zou et al., 2013; Chen, 2014; Huang et al., 2014; Liu, 2014; Wang, 2014; Zhao, 2014; Hou, 2015; Ji, 2015; Tan, 2015; Wang et al., 2015; Ding, 2016; Miao et al., 2016; Pan, 2016; Wang, 2016; Xia, 2016; Peng et al., 2017; Liu, 2018; Lv, 2018; Wang, 2018; Wang et al., 2018; Zhao, 2018; Zhao et al., 2018; Liao and Huang, 2019; Chen and Chen, 2020; Ge et al., 2020; Wang ang Zhu, 2020; Gao and Chen, 2021; Xia et al., 2021; Xie and Huang, 2021; Zhao and Xie, 2021; Zhou, 2021; Pan et al., 2022; Yang, 2022; Zhao et al., 2022).

### 3.2 Basic features of literature research

Table 1 shows the basic information of the included studies. All of the trials were double-arm RCTs. All of the studies were operated in China. One study was published in English (Ge et al., 2020), and the others were written in Chinese. A total of 6,694 patients were randomly divided into an SBP group and a control group, including 4,256 men. The mean age of the participants ranged from 51.6 to 81.7 years, who can be defined as middle-aged and elderly patients. Sample sizes range from 30 participants per arm to 1,335 people per arm. In all included studies, the standard type, and dose of CWM in the SBP treatment group were the same as those in the control group. The ingredients of SBP in all studies were the same, and the dosage of SBP in the experimental group was 22.5–67.5 mg three times a day. The shortest intervention period was 2 weeks while the longest was 24 months, and 3 months were the main ones (11/42, 26.19%). Three trials provided specific follow-up after treatment, with a maximum follow-up of 24 months (Peng et al., 2017; Ge et al., 2020) and a minimum follow-up of 6 months (Gao and Chen, 2021). A total of three studies received funding support (Tan, 2015; Ge et al., 2020; Ge et al., 2020; Zhao et al.). All the studies examined the effect of SBP combined with CWM on SCAD, six studies (Yang et al., 2013; Ding, 2016; Peng et al., 2017; Ge et al., 2020; Gao and Chen, 2021; Zhao and Xie, 2021) for MACE, twenty-seven studies (Sun, 2010; Xu et al., 2010; Shi and Hang, 2011; Guo and Tan, 2012; Sun, 2013; Chen, 2014; Huang et al., 2014; Liu, 2014; Wang, 2014; Hou, 2015; Ji, 2015; Tan, 2015; Wang et al., 2015; Ding, 2016; Wang, 2016; Xia, 2016; Liu, 2018; Lv, 2018; Wang et al., 2018; Zhao, 2018; Zhao et al., 2018; Chen and Chen, 2020; Zhu, 2020; Gao and Chen, 2021; Wang ang; Xia et al., 2021; Pan et al., 2022; Zhao et al., 2022) for the total effective rate of angina symptom improvement, sixteen studies (Sun, 2010; Xu et al., 2010; Guo and Tan, 2012; Sun, 2013; Liu, 2014; Wang, 2014; Zhao, 2014; Hou, 2015; Ji, 2015; Xia, 2016; Lv, 2018; Wang et al., 2018; Zhao et al., 2018; Wang ang Zhu, 2020; Gao and Chen, 2021; Pan et al., 2022) for ECG improvement, eighteen studies (Xu et al., 2010; Guo and Tan, 2012; Sun, 2013; Zou et al., 2013; Wang, 2014; Chen, 2014; Zhao, 2014; Hou, 2015; Ji, 2015; Pan, 2016; Xia, 2016; Peng et al., 2017; Lv, 2018; Ge et al., 2020; Wang ang Zhu, 2020; Liu, 2018; Gao and Chen, 2021; Zhao and Xie, 2021) for AEs. Supplementary Material S5 provided follow-up times for all outcome measures.

**TABLE 1:** Characteristics of the included trials.

Study ID	Sample size	Gender	Age (year)		Angina classification(CCS)	Duration	Interventions		SBP dosage	Outcomes
	(T/C)	(M/F)	T	C			T	C		
Ge et al., 2020	1,335/1,327	1886/776	63.9 ± 9.8	63.7 ± 9.9	Ⅰ∼Ⅲ	24 months	SBP + CWM	CWM + placebo	45mg, t.i.d	①④
Yang, (2022)	40/40	50/30	71.53 ± 5.06	73.38 ± 5.47	Ⅰ∼Ⅳ	3 months	SBP + CWM	CWM	67.5mg, t.i.d	⑤⑥⑦
Zhao et al., 2022	54/54	59/49	62.1 ± 10.4	62.5 ± 10.8	Ⅰ∼Ⅲ	1 month	SBP + CWM	CWM	45mg, t.i.d	②⑤⑥⑧
Pan et al., 2022	68/65	70/63	74.23 ± 8.47	76.87 ± 7.69	Ⅰ∼Ⅳ	1 month	SBP + CWM	CWM	45mg, t.i.d	②③⑤⑧
Zhou, (2021)	35/35	41/29	71.33 ± 11.20	71.23 ± 11.12	NR	2 weeks	SBP + CWM	CWM	67.5mg, t.i.d	⑤⑥
Zhao and Xie, (2021)	75/75	79/71	72.3 ± 11.5	72.4 ± 11.9	Ⅰ∼Ⅳ	6 months	SBP + CWM	CWM	45mg, t.i.d	①④
Xie and Huang, (2021)	39/39	43/35	61.38 ± 5.74	61.19 ± 5.62	Ⅰ∼Ⅳ	2 months	SBP + CWM	CWM	67.5mg, t.i.d	⑤⑥
Xia et al., 2021	61/61	74/48	65.53 ± 8.67	65.53 ± 8.67	Ⅰ∼Ⅲ	3 months	SBP + CWM	CWM	67.5mg, t.i.d	②⑤⑥⑧
Gao and Chen, (2021)	78/78	83/73	63 ± 12	63 ± 12	Ⅰ∼Ⅳ	3 months	SBP + CWM	CWM	45mg, t.i.d	①②③④
Wang and Zhu, (2020)	40/40	52/28	70.5 ± 9.5	71.2 ± 9.8	Ⅰ∼Ⅲ	2 weeks	SBP + CWM	CWM	67.5mg, t.i.d	②③④
Chen and Chen, (2020)	44/44	56/32	65.09 ± 7.52	65.33 ± 7.40	Ⅰ∼Ⅲ	3 months	SBP + CWM	CWM	22.5–45mg, t.i.d	②⑤⑥
Zhao et al., 2018	40/40	41/39	58.80 ± 8.38	56.53 ± 8.93	Ⅰ∼Ⅳ	2 months	SBP + CWM	CWM	45mg, t.i.d	②③
Zhao, (2018)	45/45	50/40	55.16 ± 5.83	54.92 ± 5.08	Ⅰ∼Ⅲ	6 months	SBP + CWM	CWM	45mg, t.i.d	②
Wang et al., 2018	30/30	24/36	55.00 ± 7.53	60.00 ± 4.88	Ⅰ∼Ⅳ	6 months	SBP + CWM	CWM	45mg, t.i.d	②③
Liu, (2018)	79/79	85/73	56.4 ± 2.3	56.9 ± 2.4	Ⅰ∼Ⅳ	2 months	SBP + CWM	CWM	67.5mg, t.i.d	②④
Peng et al., 2017	60/54	65/49	60.56 ± 3.53	61.65 ± 4.75	Ⅰ∼Ⅲ	24 months	SBP + CWM	CWM + placebo	45mg, t.i.d	①④⑤
Xia, (2016)	40/40	52/28	61.0 ± 7.5	60.2 ± 8.7	NR	6 months	SBP + CWM	CWM	45mg, t.i.d	②③④
Wang, (2016)	45/45	62/28	65.46 ± 5.53	66.35 ± 6.57	Ⅰ∼Ⅳ	12 months	SBP + CWM	CWM	45mg, t.i.d	②⑧
Ding, (2016)	42/42	50/34	59 ± 2.3	60 ± 2.2	Ⅰ∼Ⅲ	6 months	SBP + CWM	CWM	45mg, t.i.d	①②
Wang et al., 2015	40/40	47/33	68.3 ± 5.2	68.3 ± 5.2	NR	1 month	SBP + CWM	CWM	45mg, t.i.d	②⑤
Tang, (2015)	84/82	92/74	67.54 ± 4.27	67.09 ± 4.45	NR	3 months	SBP + CWM	CWM	45mg, t.i.d	②⑤
Ji, (2015)	34/34	33/35	61.21 ± 9.17	62.23 ± 8.87	Ⅰ∼Ⅲ	2 months	SBP + CWM	CWM	45mg, t.i.d	②③④⑤⑥
Hou, (2015)	34/34	49/19	71 ± 1.6	72 ± 1.2	Ⅰ∼Ⅲ	2 months	SBP + CWM	CWM	45mg, t.i.d	②③④
Zhao, (2014)	60/60	66/54	63 ± 8.5	64 ± 4.5	Ⅰ∼Ⅳ	6 months	SBP + CWM	CWM	45mg, t.i.d	③④⑧
Wang, (2014)	55/50	61/44	81.7 ± 6.03 (total)		Ⅰ∼Ⅳ	3 months	SBP + CWM	CWM	45mg, t.i.d	②③④
Liu, (2014)	30/30	35/25	60.5 (total)		Ⅰ∼Ⅳ	3 months	SBP + CWM	CWM	45mg, t.i.d	②③
Huang et al., 2014	60/60	71/49	66.31 ± 5.25	66.75 ± 6.25	Ⅱ∼Ⅲ	1 month	SBP + CWM	CWM	45mg, t.i.d	②
Chen, 2014	45/45	52/38	57.3 ± 2.1	58.1 ± 1.8	Ⅰ∼Ⅳ	2 months	SBP + CWM	CWM	45–67.5mg, t.i.d	②④
Zou et al., 2013	54/54	60/48	64.5 ± 9.6 (total)		Ⅰ∼Ⅳ	3 months	SBP + CWM	CWM	45mg, t.i.d	④
Yang et al., 2013	45/45	46/44	62.7 ± 10.4	61.4 ± 10.3	Ⅰ∼Ⅳ	1 month	SBP + CWM	CWM	45mg, t.i.d	①
Sun, (2013)	56/56	65/57	51.6	51.7	Ⅰ∼Ⅳ	2 months	SBP + CWM	CWM	22.5–45mg, t.i.d	②③④
Lv, (2018)	32/32	38/26	56.3 ± 4.9 (total)		Ⅰ∼Ⅳ	2 months	SBP + CWM	CWM	45mg, t.i.d	②③④⑧
Guo and Tan, (2012)	58/56	99/15	76.3	74.6	NR	6 weeks	SBP + CWM	CWM	45mg, t.i.d	②③④⑤⑥
Shi and Hang, (2011)	65/62	79/48	56.83 ± 11.89	55.26 ± 11.00	Ⅱ∼Ⅲ	2 weeks	SBP + CWM	CWM	45mg, t.i.d	②
Xu et al., 2010	42/41	46/37	67.54 ± 4.27	67.09 ± 4.45	Ⅰ∼Ⅳ	3 months	SBP + CWM	CWM	45mg, t.i.d	②③④⑤⑦⑧
Sun, (2010)	50/50	73/27	58.5	57.2	NR	3 months	SBP + CWM	CWM	45–67.5mg, t.i.d	②③
Wang, (2018)	51/51	65/37	65.0 ± 6.0	65.3 ± 6.2	NR	2 months	SBP + CWM	CWM	45mg, t.i.d	⑦
Miao et al., 2016	40/38	42/36	67 ± 9	65 ± 12	NR	2 months	SBP + CWM	CWM	45mg, t.i.d	⑦⑧
Liao and Huang (2019)	47/47	61/33	70.67 ± 7.95	71.24 ± 8.13	NR	6 months	SBP + CWM	CWM	45mg, t.i.d	⑦
Pan, (2016)	45/45	50/40	46.4 ± 5.2	43.7 ± 4.5	NR	1 month	SBP + CWM	CWM	45mg, t.i.d	⑦④
Wu et al., 2012	44/44	49/39	64	63	NR	3 months	SBP + CWM	CWM	45mg, t.i.d	⑦
Jiang, (2012)	42/42	55/29	72.6	73.5	NR	6 months	SBP + CWM	CWM	45mg, t.i.d	⑦

T, trial group; C, control group; NR, not report; SBP, shexiang baoxin pill; CWM, the conventional western medicine (antiplatelet drugs, lipid-lowering drugs, vasodilators of nitrate, and other conventional western medicine recommended by the guideline); t. i.d., three times a day; CCS, canadian cardiovascular society; Outcomes: ①major adverse cardiovascular events, ②the total effective rate of angina symptom improvement, ③electrocardiogram improvement, ④adverse events,⑤angina pectoris frequency, ⑥angina pectoris duration, ⑦left ventricular ejection fraction, ⑧blood lipid index.

### 3.3 Literature quality assessment

Supplementary Material S6 shows the risk of bias of the included studies for each outcome from low to high risk. All 42 studies referred to randomization, of which 12 used the random number table (Wang, 2016; Peng et al., 2017; Liu, 2018; Wang, 2018; Liao and Huang, 2019; Chen and Chen, 2020; Wang and Zhu, 2020; Gao and Chen, 2021; Ge, 2021; Xia et al., 2021; Xie and Huang, 2021; Pan et al., 2022), and the remaining 30 studies did not specifically report randomization method. Only one trial (Ge, 2021) adequately reported allocation concealment details. Three studies (Wang, 2016; Peng et al., 2017; Ge, 2021) reported the use of double-blinding, which was considered a low risk of “bias due to deviations from intended interventions”. For the total effective rate of angina symptom improvement, one study (Zhao, 2018) included 90 subjects, but only 88 subjects had outcome data, and no reason was explained, with a high risk of “bias due to miss outcome data”. Due to the objectivity of the outcome indicators, some results have no or little room for judgment, and “bias in the selection of the reported result” of LVEF and blood lipid level should be considered “low risk”. In addition, we found that one of the studies included in this review (Ge, 2021) had a low risk of “bias in the selection of the reported result” because the methods of outcome measurement and analysis were consistent with the prespecified protocol, whereas the remaining 41 studies did not find pre-specified study protocols and were assessed as “some concerns” of “bias in the selection of the reported result”.

### 3.4 Primary outcome measures

#### 3.4.1 MACE

**FIGURE 2:**
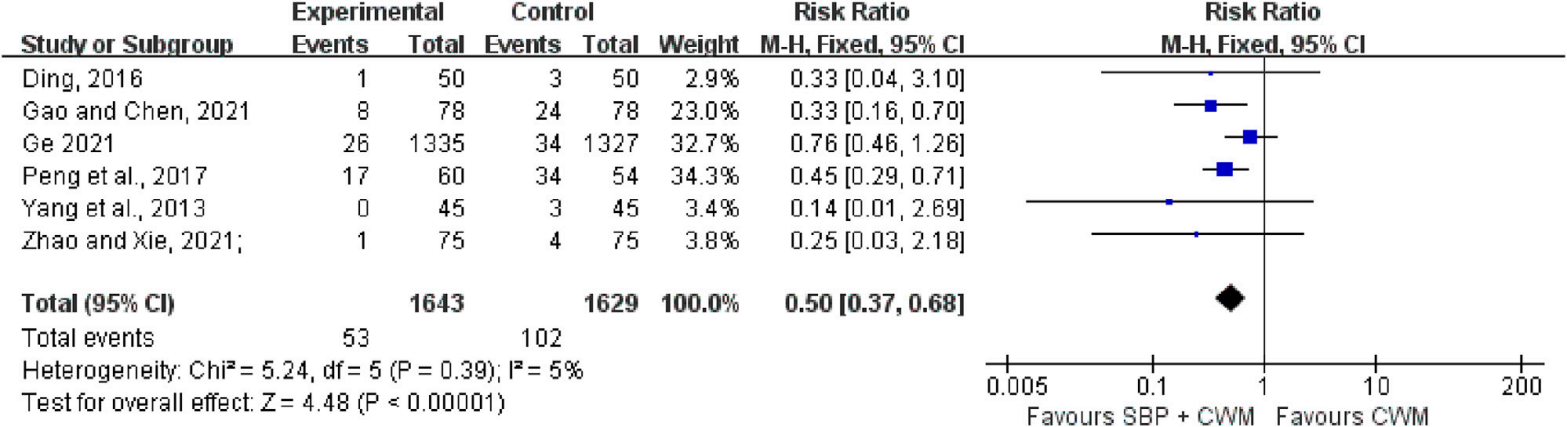
Forest plot of major adverse cardiovascular events.

Six trials with 3,272 patients reported the occurrence of MACE. There was little statistical heterogeneity among the studies (*I* ^2^ = 5%, *p* = 0.39), and a fixed-effect model was used for meta-analysis. The results indicated that the experimental group (SBP plus CWM) had better efficacy in lowering the incidence of MACE compared with the control group (RR = 0.50, 95% CI: 0.37 to 0.68, *p* < 0.00001; Figure 2).

#### 3.4.2 Angina symptom improvement

**FIGURE 3:**
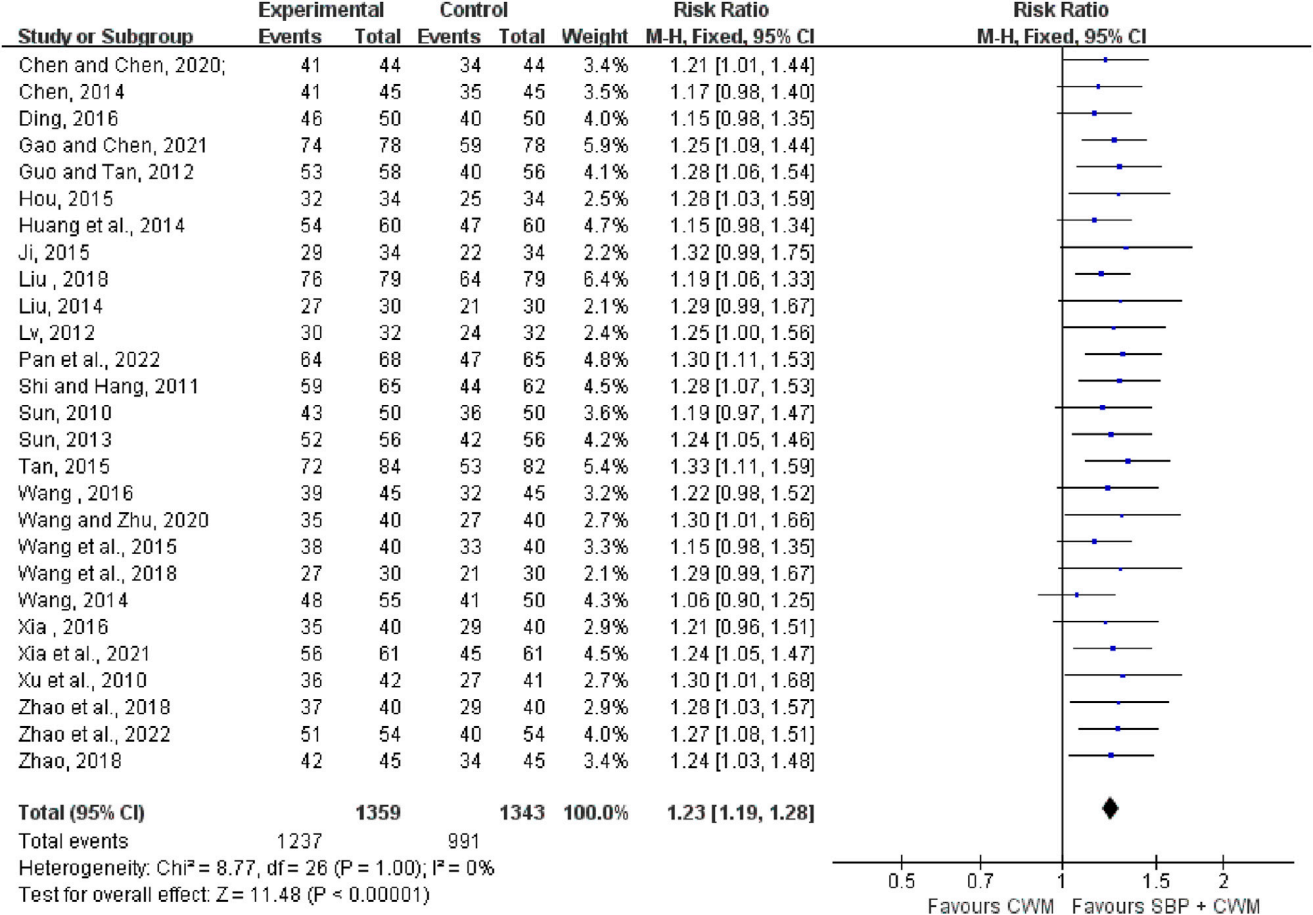
Forest plot of the total effective rate of angina symptom improvement.

Twenty-seven trials with 2,702 patients reported the total effective rate of angina symptom improvement. The meta-analysis indicated that SBP therapy significantly improved the total effective rate of angina symptom improvement showing a compelling homogeneity (RR = 1.23, 95% CI: 1.19 to 1.28, *p* < 0.00001; *I* ^2^ = 0%; Figure 3).

#### 3.4.3 ECG improvement

**FIGURE 4:**
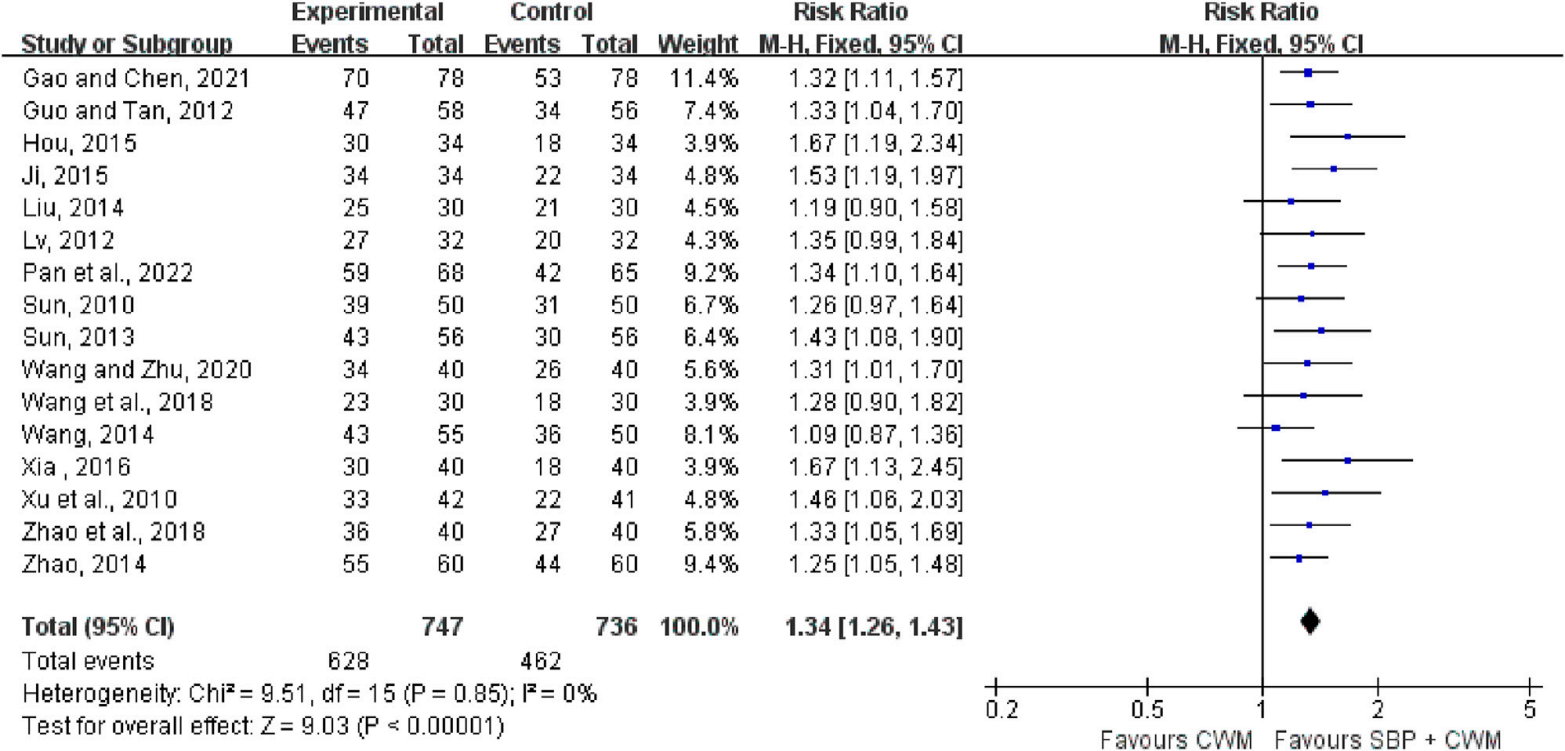
Forest plot of electrocardiogram improvement.

Sixteen trials with 1,483 cases reported the effective rate of ECG improvement. The results showed that compared with the control group, the experimental group could significantly increase the effective rate of ECG improvement (RR = 1.34, 95% CI: 1.26 to 1.43, *p* < 0.00001; *I* ^2^ = 0%), see Figure 4 for details.

#### 3.4.4 AEs

**FIGURE 5:**
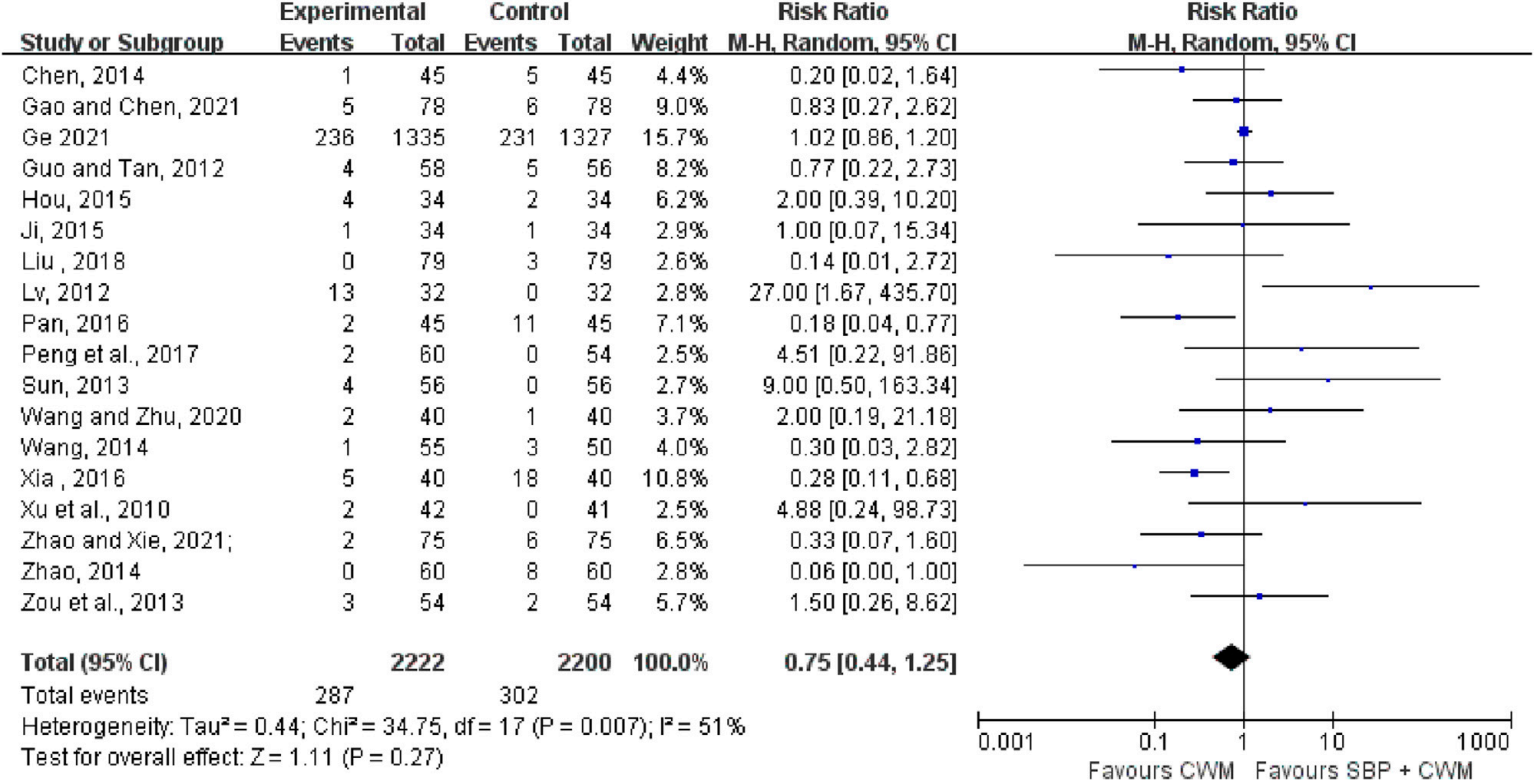
Forest plot of adverse events.

Eighteen studies documented AEs in a total of 4,422 patients. Compared with CWM, the SBP group did not increase the risk of AEs (RR = 0.75, 95% CI: 0.44 to 1.25, *p* = 0.27; *I* ^2^ = 51%; Figure 5) suggesting SBP therapy was safe. Our results revealed that gastrointestinal discomfort, tongue numbness, headache, and rash constitute the most frequently occurring AEs. Remarkable adverse reactions were modest, with no severe adverse impacts, detailed information is shown in Supplementary Material S7.

### 3.5 Secondary outcome measures

#### 3.5.1 Angina pectoris frequency

**FIGURE 6:**
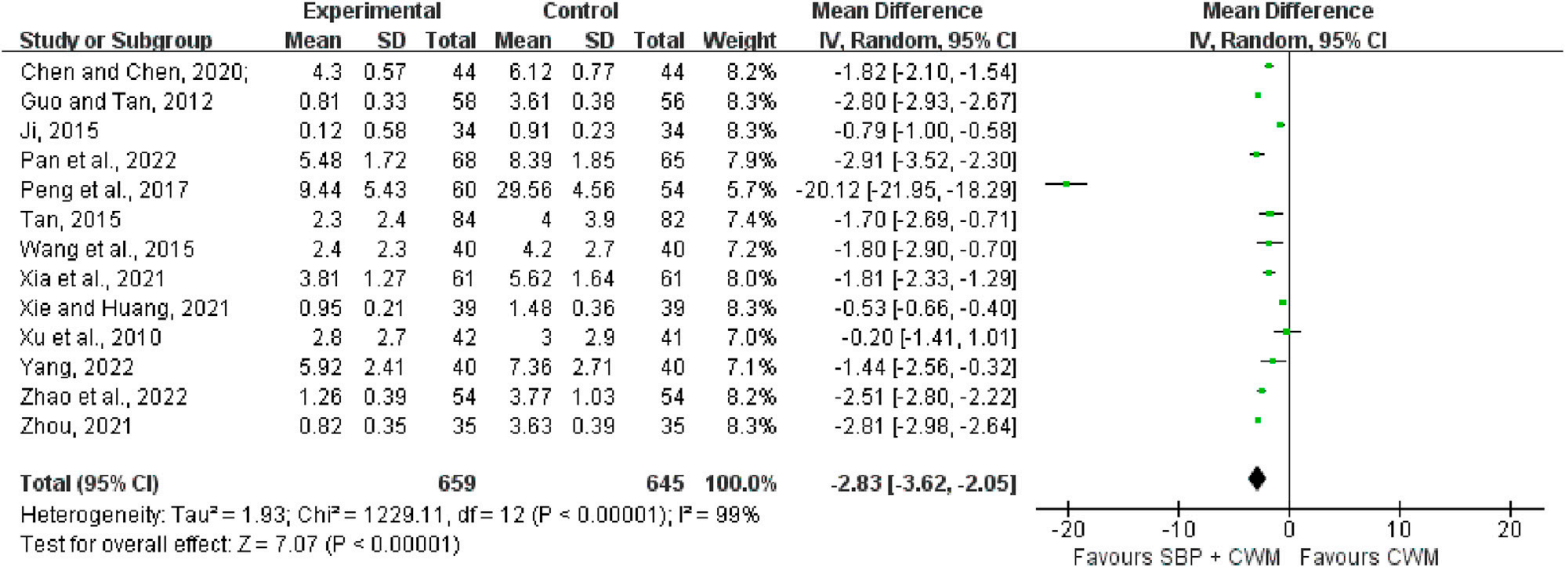
Forest plot of angina pectoris frequency.

Thirteen studies covering 1,304 patients included angina pectoris frequency as the outcome. The merged data indicated that the combination of SBP was more effective than CWM alone in reducing the frequency of angina pectoris (MD = −2.83, 95% CI: −3.62 to −2.05, *p* < 0.00001; *I* ^2^ = 99%; Figure 6).

#### 3.5.2 Angina pectoris duration

**FIGURE 7:**
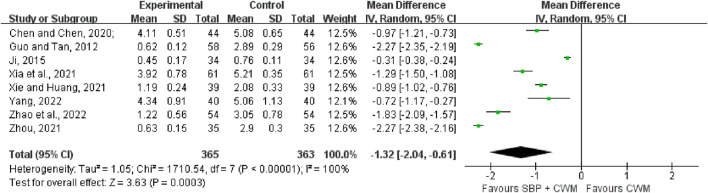
Forest plot of angina pectoris duration.

As shown in Figure 7, eight studies (728 patients) were included to compare the differences between the experimental group and the control group for the duration of angina pectoris. The results of the heterogeneity test showed that *I* ^*2*^ = 100%, *p* < 0.00001, indicating a high degree of heterogeneity among the studies, so a random-effects model was used for the analysis. The results of the Meta-analysis indicated that compared with the control group, the experimental group could effectively shorten the duration of angina pectoris in patients with SCAD (MD = −1.32, 95% CI: −2.04 to −0.61, *p* = 0.0003).

#### 3.5.3 Left ventricular ejection fraction

**FIGURE 8:**
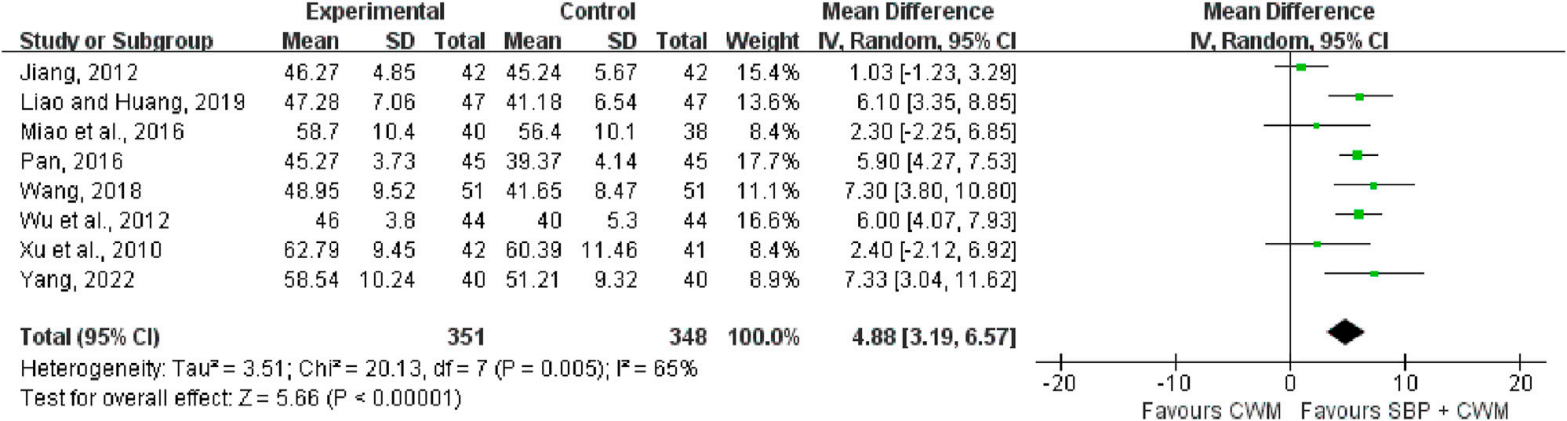
Forest plot of left ventricular ejection fraction.

Eight studies covering 699 patients included LVEF (Figure 8) as the outcome. Compared with CWM, the combination of SBP showed a higher increase in LVEF (MD = 4.88, 95% CI: 3.19 to 6.57, *p* < 0.00001; *I* ^2^ = 65%).

#### 3.5.4 Blood lipid level

**FIGURE 9:**
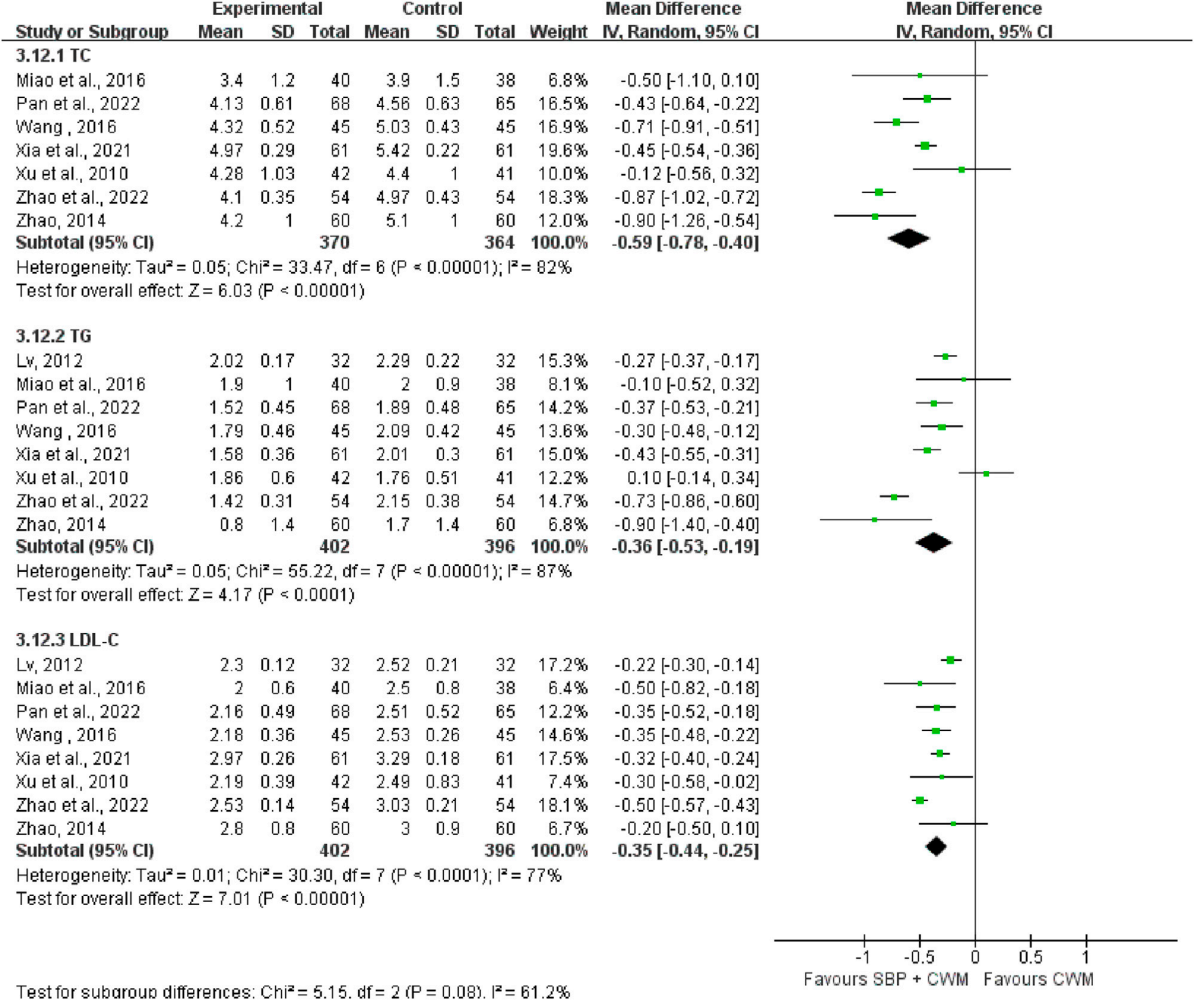
Forest plots of total cholesterol, triglyceride, and low-density lipoprotein cholesterol.

**FIGURE 10:**
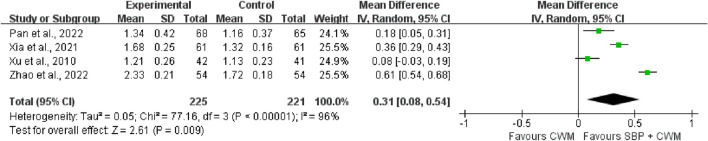
Forest plot of high-density lipoprotein cholesterol.

Blood lipid level was measured with TC, TG, LDL-C, and HDL-C. Seven studies covering 734 patients reported SBP therapy reduced TC (MD = −0.59, 95% CI: −0.78 to −0.40, *p* < 0.00001; *I* ^2^ = 82%; Figure 9). Eight studies reported TG, containing 798 patients. Compared with CWM, SBP treatment showed a decrease in TG (MD = −0.36, 95% CI: −0.53 to −0.19, *p* < 0.00001; *I* ^2^ = 87%; Figure 9). LDL-C was reported in eight studies (MD = −0.35, 95% CI: −0.44 to −0.25, *p* < 0.00001; *I* ^2^ = 77%; Figure 9) indicating SBP treatment substantially lowered LDL-C. HDL-C was recorded in four trials with the improvement in the experimental group (MD = 0.31, 95% CI: 0.08 to 0.54, *p* = 0.009; *I* ^2^ = 96%; Figure 10).

### 3.6 Meta-regression and subgroup analysis

As results of angina pectoris frequency, angina pectoris duration, LVEF, TC, TG, LDL-C, and HDL-C showed high heterogeneity in our study, we performed a meta-regression analysis based on publication year, mean age, duration of treatment, and sample size. The results of meta-regression analysis showed that the duration of treatment and sample size were significant sources of heterogeneity for angina pectoris duration, and TG, respectively (*p* < 0.05). In addition, the results revealed that publication year, mean age, treatment duration, and sample size were not remarkable sources of heterogeneity for angina pectoris frequency, LVEF, TC, LDL-C, and HDL-C (all *p* > 0.05; Supplementary Material S8).

The intervention course of the drug was crucial to the clinical efficacy. We used subgroup analyses to determine whether treatment effects differed by treatment duration. As shown in Table 2 and Supplementary Material S9, the results of subgroup analysis were consistent with the overall study results. Regardless of the length of the course of treatment, SBP combined with CWM could significantly improve the incidence of angina pectoris frequency, angina pectoris duration, LVEF, and blood lipid level. We also found that with the prolongation of the course of treatment, the benefit of some indicators was more obvious, and the combined effect size was larger, such as TC, TG, and LDL-C. In addition, for some outcomes, heterogeneity was reduced after the subgroup analysis, such as angina pectoris frequency, angina pectoris duration, LVEF, TC, and LDL-C, suggesting that treatment duration may be the source of heterogeneity. However, it should be noted that there is still considerable heterogeneity in the remaining subgroups, suggesting that heterogeneity may come from other sources. We believed that the differences in detection equipment and techniques used in different studies may be one of the main sources.

**TABLE 2:** Results of subgroup analysis.

Outcome or subgroup	Studies	Participants	MD/RR (95%CI)	*Z*	*P*	Heterogeneity	
						*I* ^2^	*P*
1. Adverse Events	18	4,422	0.75 (0.44, 1.25)	1.11	0.27	51%	0.007
Treatment duration							
≤2 months	9	844	0.99 (0.35, 2.83)	0.02	0.98	57%	0.02
3 months	4	452	0.95 (0.41, 2.21)	0.12	0.91	0%	0.48
6 months	3	350	0.26 (0.12, 0.55)	3.54	0.0004	0%	0.53
12 months	2	2,776	1.02 (0.87, 1.20)	0.24	0.81	0%	0.33
2. Angina Pectoris Frequency	13	1,304	−2.83 (−3.62, −2.05)	7.07	<0.00001	99%	<0.00001
Treatment duration							
<2 months	5	505	−2.74 (−2.89, −2.58)	34.84	<0.00001	39%	0.16
2 months	2	146	−0.65 (−0.90, −0.39)	5.00	<0.00001	76%	0.04
3 months	5	539	−1.62 (−2.02, −1.22)	7.95	<0.00001	42%	0.14
24 months	1	114	−20.12 (−21.95, −18.29)	21.49	<0.00001	—	—
3. Angina Pectoris Duration	8	728	−1.32 (−2.04, −0.61)	3.63	0.0003	100%	<0.00001
Treatment duration							
<2 months	3	292	−2.17 (−2.34, −2.00)	24.56	<0.00001	81%	0.005
2 months	2	146	−0.60 (−1.17, −0.03)	2.06	0.04	98%	<0.00001
3 months	3	290	−1.04 (−1.34, −0.73)	6.68	<0.00001	71%	0.03
4. LVEF	8	699	4.88 (3.19, 6.57)	5.66	<0.00001	65%	0.005
Treatment duration							
≤2 months	3	270	5.65 (3.58, 7.72)	5.34	<0.00001	33%	0.22
3 months	3	251	5.59 (3.41, 7.77)	5.03	<0.00001	26%	0.26
6 months	2	178	3.50 (−1.47, 8.47)	1.38	0.17	87%	0.005
5. TC	7	734	−0.59 (−0.78, −0.40)	6.03	<0.00001	82%	<0.00001
Treatment duration							
≤2 months	3	319	−0.63 (−0.98, −0.27)	3.44	0.0006	83%	0.003
3 months	2	205	−0.36 (−0.65, −0.07)	2.40	0.02	52%	0.15
≥6 months	2	210	−0.75 (−0.93, −0.58)	8.56	<0.00001	0%	0.36
6. TG	8	798	−0.36 (−0.53, −0.19)	4.17	<0.0001	87%	<0.00001
Treatment duration							
1 month	2	241	−0.55 (−0.91, −0.20)	3.07	0.002	92%	0.0006
2 months	2	142	−0.26 (−0.36, −0.17)	5.46	<0.00001	0%	0.44
3 months	2	205	−0.18 (−0.69, 0.34)	0.66	0.51	93%	<0.0001
≥6 months	2	210	−0.55 (−1.13, 0.03)	1.87	0.06	79%	0.03
7. LDL-C	8	798	−0.35 (−0.44, −0.25)	7.01	<0.00001	77%	<0.0001
Treatment duration							
1 month	2	241	−0.45 (−0.59, −0.31)	6.22	<0.00001	61%	0.11
2 months	2	142	−0.32 (−0.58, −0.06)	2.38	0.02	65%	0.09
3 months	2	205	−0.32 (−0.39, −0.24)	8.18	<0.00001	0%	0.89
≥6 months	2	210	−0.33 (−0.45, −0.21)	5.37	<0.00001	0%	0.37
8. HDL-C	4	446	0.31 (0.08, 0.54)	2.61	0.009	96%	<0.0001
Treatment duration							
1 month	2	241	0.40 (−0.02, 0.82)	1.86	0.06	97%	<0.00001
3 months	2	205	0.22 (−0.05, 0.50)	1.59	0.11	94%	<0.00001

CI, confidence interval; MD, mean difference; RR, risk ratio; LVEF, left ventricular ejection fraction; TC, total cholesterol; TG, triglyceride; LDL-C, low-density lipoprotein cholesterol; HDL-C, high-density lipoprotein cholesterol.

### 3.7 Sensitivity analysis and publication bias

The main outcomes, encompassing MACE, the total effective rate of angina symptom improvement, ECG improvement, and AEs were tested by the sensitivity analysis, which involved removing each trial in turn to assess the robustness of the main outcome. The pooled RR values of MACE, the total effective rate of angina symptom improvement, and ECG improvement were relatively stable and reliable, according to Supplementary Material S10. However, one study (Ge, 2021) had a certain impact on the stability of the results of adverse events, which may be related to the study’s high quality, multiple centers, wide population, and long follow-up.

Publication bias was conducted on the outcomes in which trials were over ten. Egger’s test was conducted to confirm the publication bias. As shown in Supplementary Material S11, the results showed ECG improvement (*p* = 0.073), AEs (*p* = 0.585), and angina pectoris frequency (*p* = 0.438) were reliable. Egger’s test of the total effective rate of angina symptom improvement demonstrated that the *p*-value was less than 0.05 (*p* = 0.035), which indicated that there is publication bias.

### 3.8 GRADE assessment

Using GRADE (Table 3), we judged the certainty in our estimates to be low across primary outcomes. For MACE, and ECG improvement, we downgraded the evidence by one level for serious risk of bias, and the evidence was judged as moderate certainty. For the total effective rate of angina symptom improvement, we downgraded the evidence by two levels owing to the serious risk of bias and publication bias, and the evidence was judged as low certainty. For AEs, angina pectoris frequency, angina pectoris duration, LVEF, and blood lipid indicators, we downgraded the evidence by two levels owing to the serious risk of bias and heterogeneity, and the evidence was judged as low certainty.

**TABLE 3:** GRADE evidence profile.

Quality assessment						No of patients		RR/MD (95% CI)	Quality	Importance
No. Of studies	Risk of bias	Inconsistency	Indirectness	Imprecision	Publication bias	SBP combined with CWM	CWM			
MACE										
6	serious^a^	no serious	no serious	no serious	none	53/1,643 (3.23%)	102/1,629 (6.26%)	RR = 0.50 (0.37, 0.68)	⊕⊕⊕O moderate	CRITICAL
Angina Symptom Improvement										
27	serious^a^	no serious	no serious	no serious	Presence^b^	1,237/1,359 (91.02%)	991/1,343 (73.79%)	RR = 1.23 (1.19, 1.28)	⊕⊕OO low	CRITICAL
ECG Improvement										
16	serious^a^	no serious	no serious	no serious	none	628/747 (84.07%)	462/736 (62.77%)	RR = 1.34 (1.26, 1.43)	⊕⊕⊕O moderate	CRITICAL
Adverse Events										
18	serious^a^	serious^c^	no serious	no serious	none	287/2,222 (12.92%)	302/2,200 (13.73%)	RR = 0.75 (0.44, 1.25)	⊕⊕OO low	CRITICAL
Angina Pectoris Frequency										
13	serious^a^	serious^c^	no serious	no serious	none	659	645	MD = −2.83 (−3.62, −2.05)	⊕⊕OO low	CRITICAL
Angina Pectoris Duration										
8	serious^a^	serious^c^	no serious	no serious	none	365	363	MD = −1.32 (−2.04, −0.61)	⊕⊕OO low	IMPORTANT
LVEF										
8	serious^a^	serious^c^	no serious	no serious	none	351	348	MD = 4.88 (3.19, 6.57)	⊕⊕OO low	IMPORTANT
TC										
7	serious^a^	serious^c^	no serious	no serious	none	370	364	MD = −0.59 (−0.78, −0.40)	⊕⊕OO low	IMPORTANT
TG										
8	serious^a^	serious^c^	no serious	no serious	none	402	396	MD = −0.36 (−0.53, −0.19)	⊕⊕OO low	IMPORTANT
**LDL-C**										
8	serious^a^	serious^c^	no serious	no serious	none	402	396	MD = −0.35 (−0.44, −0.25)	⊕⊕OO low	IMPORTANT
HDL-C										
4	serious^a^	serious^c^	no serious	no serious	none	225	221	MD = 0.31 (0.08, 0.54)	⊕⊕OO low	IMPORTANT

SBP, shexiang baoxin pill; CWM, the conventional western medicine; CI, confidence interval; MD, mean difference; RR, risk ratio;

^a^ Random protocol, blinding, and allocation concealment of some studies were not clear;

^b^ Quantitative evaluation of the included data indicated publication bias;

^c^ Heterogeneity (*I* ^2^ > 50%, *p* < 0.05) was found.

### 3.9 Trial sequential analysis

**FIGURE 11:**
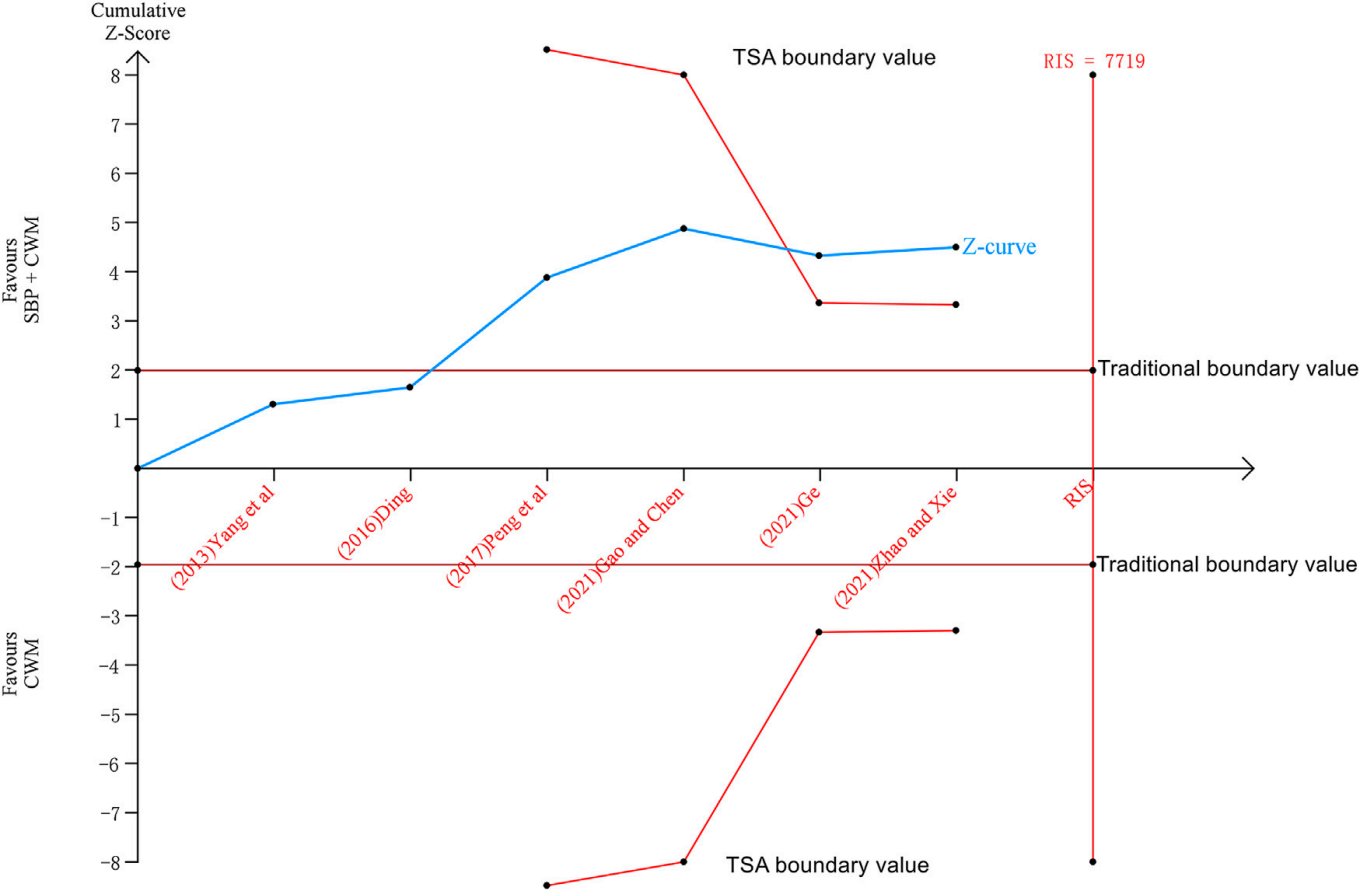
Trial sequential analysis of major adverse cardiovascular events.

We performed the trial sequential analysis of six trials reporting MACE. The parameters of this study were set as follows: type I error probability *α* = 5%, statistical power 1-β = 80%, and RR reduced by 20%. The sample size was used as the required information size (RIS) for the two-sided test. The results show that the cumulative Z value after the fifth study (Ge et al., 2020) has crossed the traditional boundary value and the TSA boundary value. Although the cumulative amount of information has not reached the required information size (RIS = 7,719), more experiments are not needed, and a positive conclusion can be obtained in advance, as shown in Figure 11.

## 4 Discussion

Generally, SCAD progresses more slowly and is milder than acute coronary syndrome. Notably, there is a common misunderstanding that the management and treatment of SCAD are mature. The reality is that the rate of misdiagnosis and underdiagnosis of SCAD is high, the quality of life of patients is reduced due to angina pectoris, the application of secondary preventive measures is insufficient, pharmacological treatment is inadequate, the patient benefit is not evident and medical costs increase, resulting in a significant number of patients converting to ACS and there is still a considerable residual cardiovascular risk (De Luca et al., 2019). In the treatment of coronary artery disease, Chinese medicine has played an important role (Liang and Gu, 2021). As a modern Chinese patent medicine, SBP has become the main supplementary drug for secondary prevention of SCAD patients in China, but there has been a lack of high-quality clinical evidence on the effect of SBP on adverse cardiovascular events in patients with SCAD and the safety of long-term use of SBP. In 2018, the “Chinese Expert Consensus on Shexiang Baoxin Pills for the Treatment of Coronary Heart Disease and Angina Pectoris” was officially published (Cardiovascular Diseases Professional Committee of Chinese Association of Integrated Traditional and Western Medicine, 2018), which systematically reviewed and summarized the pharmacological effects, clinical efficacy and safety of SBP, and strongly promoted the clinical application of SBP. With the deepening of clinical practice and research, more and more high-quality research evidence of SBP has been published. Therefore, it is necessary to conduct a systematic review and meta-analysis on the efficacy and safety of SBP in the treatment of SCAD, incorporate more strong evidence, and further clarify the applicable population and the strength of the evidence, to better guide the clinical application of SBP.

### 4.1 Summary of evidence

Our meta-analysis evaluated the clinical efficacy and safety of SBP in patients with SCAD through the 42 included studies with a total of 6,694 patients, which is the first study focusing on SBP treatment for SCAD patients. The results indicated that SBP combined with CWM could improve the incidence of MACE, the total effective rate of angina symptom improvement and ECG improvement, angina pectoris frequency, angina pectoris duration, and LVEF suggesting the experimental group was superior in clinical efficacy. The ultimate goal of drug therapy for SCAD is to reduce mortality, improve long-term survival, decrease the incidence of important cardiovascular events, and ensure the quality of life. MACE, as the hard endpoint of clinical observation, can objectively and directly reflect drug efficacy and disease prognosis. This study showed that the incidence of MACE was 3.23% (53/1,643) in the experimental group compared with 6.26% (102/1,629) in the control group. The incidence of MACE in the SBP treatment group after treatment was 48.4% lower than that in the control group. The total effective rate of angina symptom improvement and ECG improvement, angina pectoris frequency, and angina pectoris duration can directly assess the severity of the disease and the degree of symptom relief in patients with angina pectoris. LVEF can reflect left ventricular function and provide a certain reference value for the diagnosis and prognosis. Our study found that SBP combined with CWM in the treatment of SCAD has the advantages of improving the total effective rate of angina symptom improvement and ECG improvement, angina pectoris frequency, angina pectoris duration, and LVEF. Dyslipidemia, especially elevated LDL-C, is an important risk factor for cardiovascular morbidity and mortality (Kopin and Lowenstein, 2017). Meta-analysis in this study demonstrated that SBP could remarkably improve the levels of TC, TG, LDL-C, and HDL-C in SCAD patients.

According to the SBP adverse reaction/event report of the National Adverse Drug Reaction Monitoring System (https://www.adrs.org.cn/), the adverse reactions of SBP collected from 2017 to 2021 were 346 cases (about 0.027%), 419 cases (about 0.029%), 479 cases (about 0.035%), 581 cases (about 0.040%), and 775 cases (about 0.048%), belonging to the rare range. Our study also listed AEs in trials to observe the safety of SBP in SCAD patients. Eighteen studies documented AEs in a total of 4,422 patients. According to the report, gastrointestinal discomfort, tongue numbness, headache, and rash are the highest four adverse symptoms. Other adverse cases including red face, liver and kidney damage, arrhythmia, and hypotension were also recorded. Altogether, compared with conventional treatment, SBP therapy did not increase the risk of AEs. What’s more, the results of subgroup analyses according to treatment duration are consistent with the overall study results. Our results also found that treatment duration may be the source of heterogeneity in angina pectoris frequency, angina pectoris duration, LVEF, TC, and LDL-C. Moreover, we determined that the outcomes of MACE, the total effective rate of angina symptom improvement, and ECG improvement were stable and reliable by sensitivity analysis. GRADE is a common method for assessing the certainty of clinical evidence and is widely used in the preparation and revision of guidelines and expert consensus. The overall certainty of the evidence of the outcomes exhibited moderate or low certainty with heterogeneity and methodological problems. Hence, we provide supporting evidence that, to a remarkable extent, SBP can potentially be recommended for planned use for SCAD patients.

### 4.2 Comparison with previous studies

Multiple systematic reviews and meta-analyses have demonstrated the efficacy and safety of TCM in treating stable coronary artery disease. A meta-analysis constituting high-quality articles involving 824 patients revealed that the application of TCM in the treatment of angina pectoris can improve the therapeutic effect, shorten the attack time, reduce the frequency of angina pectoris, and improve the quality of life (Chen et al., 2021), which was consistent with our results. Nevertheless, their study had no obvious reducing effect on blood lipids. The reason may be the numerous varieties of formulations and ingredients of TCM and insufficient clinical samples. A previous meta-analysis found that corn silk decoction, a Chinese medicine prescription, may improve the levels of TC, TG, and LDL-C (Shi et al., 2019). However, this study did not pay much attention to the clinical endpoints of TCM and could not provide direct evidence of prognosis. The findings of two other meta-analyses indicated that the combination of TCM significantly improved performance compared with CWM solely for the treatment of angina pectoris (Liu et al., 2016; Huiping et al., 2019). Regrettably, they both suffered from methodological quality deficiencies. A prior systematic review demonstrated that in patients with SCAD, revascularization combined with conventional therapy did not lead to an overall survival advantage over medical therapy alone (Laukkanen and Kunutsor, 2021). However, revascularization plus medical therapy may reduce the overall risk of the composite outcome of all-cause death, myocardial infarction, readmission, revascularization, or stroke. This contemporary meta-analysis highlights more effective symptomatic relief of angina pectoris with appropriate adjustment of medical therapy and invasive strategies.

### 4.3 Limitations

This review is the first attempt to focus on the efficacy and safety of SBP in the treatment of SCAD and has the strength to follow the rigorous review process of Cochrane methodology, reporting standards such as PRISMA, and addressing quality of evidence using the GRADE system. Although we tried to identify all the available evidence, this study has several limitations. Firstly, a high risk of bias existed owing to the lack of blinding and the unclear randomization methods. Secondly, substantial heterogeneity was observed in most outcomes except MACE, the total effective rate of angina symptom improvement, and ECG improvement. Subgroup analysis showed reduced heterogeneity according to treatment duration. Since SCAD was complex, etiology, disease history, nursing treatment, and western treatment strategies may all contribute to the presence of heterogeneity. More research in specific areas is needed to fully assess how these factors play a role in heterogeneity. Thirdly, GRADE evidence quality ratings are mostly low or moderate, and relevant results should be treated with caution.

### 4.4 Implications for research

We herein reveal important ideas that may advance research in this field. Firstly, it is evident that strategies that improve the methodological quality of RCTs are urgently needed. Going forward, we recommend that more high-quality RCTs should be conducted to improve the strength of the evidence, especially focusing on the implementation of subject-centered randomization, allocation concealment, and blinding. Secondly, RCTs should be reported completely and comprehensively by the CONSORT statement (Cheng, et al., 2017), with particular attention to the reporting of etiology, medical history, nursing treatment, western treatment strategies, and follow-up to find sources of heterogeneity and clarify the prognosis of SCAD patients. Thirdly, despite the revelation that SBP therapy in the analyzed studies was somewhat safe for patients with SCAD, further investigations are needed to confirm the safety of SBP for SCAD. A standard reporting format for adverse drug reactions has been developed (Bian, et al., 2010), and we propose that close attention should be paid to improving the reporting of adverse reactions of SBP. To conclusively understand the long-term safety profile of SBP in patients with SBP, clinical studies incorporating longer follow-up periods are recommended. Our results suggest that SBP combined with CWM can be an alternative treatment for SCAD patients, nonetheless, further large clinical studies should be conducted to explore the long-term safety, efficacy, and optimized dosages of SBP for treating SCAD.

## 5 Conclusion

In summary, the available evidence indicates that SBP combined with CWM may be effective in the treatment of SCAD to improve the incidence of MACE, the total effective rate of angina symptom improvement and ECG improvement, angina pectoris frequency, angina pectoris duration, LVEF, blood lipid level. However, the risk of bias in the included studies was generally low to high, and the credibility of some results was reduced by heterogeneity. Moreover, the safety of Shexiang Baoxin Pill remains uncertain, more carefully designed large-sample, long-term follow-up RCT should be carried out in the future to provide reliable evidence for SBP in treating SCAD.

## Data Availability

The original contributions presented in the study are included in the article/Supplementary Materials, further inquiries can be directed to the corresponding authors.
