# Supplementary material for: Efficacy and safety of Shexiang Baoxin Pill for stable coronary artery disease: A systematic review and meta-analysis of 42 randomized controlled trials
Source: Front Pharmacol. 2022 Nov 14;13:1002713. doi: 10.3389/fphar.2022.1002713 (PMC9701736; doi:10.3389/fphar.2022.1002713)

**Table of contents**

[Supplementary File S1. The main components of Shexiang Baoxin Pill 2](#_Toc116324991)

[Supplementary File S2. PRISMA 2020 checklist 4](#_Toc116324992)

[Supplementary File S3. Search strategies for databases. 8](#_Toc116324993)

[Supplementary File S4. A list of excluded studies by reading the full text. 10](#_Toc116324994)

[Supplementary File S5. The follow-up times for all outcome measures. 12](#_Toc116324995)

[Supplementary File S6. ROB 2.0 for outcomes. 13](#_Toc116324996)

[Supplementary File S7. Adverse drug reactions or adverse events. 19](#_Toc116324997)

[Supplementary File S8. Results of meta-regression analysis. 20](#_Toc116324998)

[Supplementary File S9. Results of subgroup analysis 21](#_Toc116324999)

[Supplementary File S10. Results of sensitivity analysis 27](#_Toc116325000)

[Supplementary File S11. Egger’s test of the outcomes. 29](#_Toc116325001)

# Supplementary File S1. The main components of Shexiang Baoxin Pill

| Formulation | Source | Species, concentration | Quality control reported  (Y/N) | Chemical analysis reported  (Y/N) |
| --- | --- | --- | --- | --- |
| Shexiang Baoxin Pill | Shanghai Hutchison Pharmaceuticals Company | - Preputial secretion of *Moschus berezovskii, M. sifanicus or M. moschiferus* [Cervidae; Moschus] concentration uncertainty - Root of *Panax ginseng C.A.Mey.*[Araliaceae; Ginseng Radix et rhizoma] concentration uncertainty - Gall-stone of *Bos taurus domesticus Gmelin*. [Bovidae; Bovis Calculus Artifactus] concentration uncertainty - Bark of *Cinnamomum cassia.* [Lauraceae; Cinnamomi Cortex]   concentration uncertainty   - Secretion from tree trunk of *Liquidambar orientalis Mill.* [Hamamelidaceae R. Br.; Styrax]   concentration uncertainty   - Secretions from skin gland of *Bufo bufo gargarizans* [Bufonidae; Bufonis Venenum]   concentration uncertainty   - Distillation and recrystallization of leaf of *Dryobalanops aromatica C.F.Gaertn.* [Lauraceae; Borneolum Syntheticum]   concentration uncertainty | Y – Prepared according to the Pharmacopoeia of China, 2020 edition | Lv, C., Chen, L., Fu, P., Yang, N., Liu, Q., Xu, Y., et al. (2017). Simultaneous quantification of 11 active constituents in Shexiang Baoxin Pill by ultraperformance convergence chromatography combined with tandem mass spectrometry. *J Chromatogr B Analyt Technol Biomed Life Sci*. 2017;1052:135-141. doi:10.1016/j.jchromb.2017.03.033.  Jiang, P., Liu, R., Dou, S., Liu, L., Zhang, W., Chen, Z., et al. (2009). Analysis of the constituents in rat plasma after oral administration of Shexiang Baoxin pill by HPLC-ESI-MS/MS. Biomed Chromatogr. 2009;23(12):1333-1343. doi:10.1002/bmc.1258. |

# Supplementary File S2. PRISMA 2020 checklist

| **Section and Topic** | **Item #** | **Checklist item** | **Location where item is reported** |
| --- | --- | --- | --- |
| **TITLE** | | |  |
| Title | 1 | Identify the report as a systematic review. | P1 |
| **ABSTRACT** | | |  |
| Abstract | 2 | See the PRISMA 2020 for Abstracts checklist. | P1-2 |
| **INTRODUCTION** | | |  |
| Rationale | 3 | Describe the rationale for the review in the context of existing knowledge. | P2-3 |
| Objectives | 4 | Provide an explicit statement of the objective(s) or question(s) the review addresses. | P3 |
| **METHODS** | | |  |
| Eligibility criteria | 5 | Specify the inclusion and exclusion criteria for the review and how studies were grouped for the syntheses. | P4 |
| Information sources | 6 | Specify all databases, registers, websites, organisations, reference lists and other sources searched or consulted to identify studies. Specify the date when each source was last searched or consulted. | P3-4 |
| Search strategy | 7 | Present the full search strategies for all databases, registers and websites, including any filters and limits used. | P3-4 |
| Selection process | 8 | Specify the methods used to decide whether a study met the inclusion criteria of the review, including how many reviewers screened each record and each report retrieved, whether they worked independently, and if applicable, details of automation tools used in the process. | P5 |
| Data collection process | 9 | Specify the methods used to collect data from reports, including how many reviewers collected data from each report, whether they worked independently, any processes for obtaining or confirming data from study investigators, and if applicable, details of automation tools used in the process. | P5 |
| Data items | 10a | List and define all outcomes for which data were sought. Specify whether all results that were compatible with each outcome domain in each study were sought (e.g. for all measures, time points, analyses), and if not, the methods used to decide which results to collect. | P4-5 |
|  | 10b | List and define all other variables for which data were sought (e.g. participant and intervention characteristics, funding sources). Describe any assumptions made about any missing or unclear information. | P4-5 |
| Study risk of bias assessment | 11 | Specify the methods used to assess risk of bias in the included studies, including details of the tool(s) used, how many reviewers assessed each study and whether they worked independently, and if applicable, details of automation tools used in the process. | P5 |
| Effect measures | 12 | Specify for each outcome the effect measure(s) (e.g. risk ratio, mean difference) used in the synthesis or presentation of results. | P5 |
| Synthesis methods | 13a | Describe the processes used to decide which studies were eligible for each synthesis (e.g. tabulating the study intervention characteristics and comparing against the planned groups for each synthesis (item #5)). | P5 |
|  | 13b | Describe any methods required to prepare the data for presentation or synthesis, such as handling of missing summary statistics, or data conversions. | P5 |
|  | 13c | Describe any methods used to tabulate or visually display results of individual studies and syntheses. | P5 |
|  | 13d | Describe any methods used to synthesize results and provide a rationale for the choice(s). If meta-analysis was performed, describe the model(s), method(s) to identify the presence and extent of statistical heterogeneity, and software package(s) used. | P5 |
|  | 13e | Describe any methods used to explore possible causes of heterogeneity among study results (e.g. subgroup analysis, meta-regression). | P5 |
|  | 13f | Describe any sensitivity analyses conducted to assess robustness of the synthesized results. | P5 |
| Reporting bias assessment | 14 | Describe any methods used to assess risk of bias due to missing results in a synthesis (arising from reporting biases). | P5 |
| Certainty assessment | 15 | Describe any methods used to assess certainty (or confidence) in the body of evidence for an outcome. | P5 |
| **RESULTS** | | |  |
| Study selection | 16a | Describe the results of the search and selection process, from the number of records identified in the search to the number of studies included in the review, ideally using a flow diagram. | P6 |
|  | 16b | Cite studies that might appear to meet the inclusion criteria, but which were excluded, and explain why they were excluded. | P6 |
| Study characteristics | 17 | Cite each included study and present its characteristics. | P6 |
| Risk of bias in studies | 18 | Present assessments of risk of bias for each included study. | P7 |
| Results of individual studies | 19 | For all outcomes, present, for each study: (a) summary statistics for each group (where appropriate) and (b) an effect estimate and its precision (e.g. confidence/credible interval), ideally using structured tables or plots. | P7-8 |
| Results of syntheses | 20a | For each synthesis, briefly summarise the characteristics and risk of bias among contributing studies. | P7-8 |
|  | 20b | Present results of all statistical syntheses conducted. If meta-analysis was done, present for each the summary estimate and its precision (e.g. confidence/credible interval) and measures of statistical heterogeneity. If comparing groups, describe the direction of the effect. | P7-8 |
|  | 20c | Present results of all investigations of possible causes of heterogeneity among study results. | P8-9 |
|  | 20d | Present results of all sensitivity analyses conducted to assess the robustness of the synthesized results. | P9 |
| Reporting biases | 21 | Present assessments of risk of bias due to missing results (arising from reporting biases) for each synthesis assessed. | P9 |
| Certainty of evidence | 22 | Present assessments of certainty (or confidence) in the body of evidence for each outcome assessed. | P9 |
| **DISCUSSION** | | |  |
| Discussion | 23a | Provide a general interpretation of the results in the context of other evidence. | P9-11 |
|  | 23b | Discuss any limitations of the evidence included in the review. | P11-12 |
|  | 23c | Discuss any limitations of the review processes used. | P11-12 |
|  | 23d | Discuss implications of the results for practice, policy, and future research. | P12 |
| **OTHER INFORMATION** | | |  |
| Registration and protocol | 24a | Provide registration information for the review, including register name and registration number, or state that the review was not registered. | P3 |
|  | 24b | Indicate where the review protocol can be accessed, or state that a protocol was not prepared. | P3 |
|  | 24c | Describe and explain any amendments to information provided at registration or in the protocol. | P3 |
| Support | 25 | Describe sources of financial or non-financial support for the review, and the role of the funders or sponsors in the review. | P13 |
| Competing interests | 26 | Declare any competing interests of review authors. | P13 |
| Availability of data, code and other materials | 27 | Report which of the following are publicly available and where they can be found: template data collection forms; data extracted from included studies; data used for all analyses; analytic code; any other materials used in the review. | P13 |

*From:*  Page MJ, McKenzie JE, Bossuyt PM, Boutron I, Hoffmann TC, Mulrow CD, et al. The PRISMA 2020 statement: an updated guideline for reporting systematic reviews. BMJ 2021;372:n71. doi: 10.1136/bmj.n71

For more information, visit: <http://www.prisma-statement.org/>

# Supplementary File S3. Search strategies for databases.

| **PubMed** | #1 " stable coronary artery disease "[MeSH Terms] OR " stable coronary heart disease "[Title/Abstract] OR " stable angina pectoris "[Title/Abstract] OR " stable angina "[Title/Abstract] OR " anginas "[Title/Abstract] OR " coronary artery disease "[Title/Abstract] OR " stable coronary "[Title/Abstract] OR " ischemic cardiomyopathy "[Title/Abstract] OR " ischaemic cardiomyopathy "[Title/Abstract] OR " ischemic CM "[Title/Abstract] OR " ischaemic CM "[Title/Abstract] OR " ischemic heart disease "[Title/Abstract] OR " ischaemic heart disease "[Title/Abstract] OR " previous myocardial infarction "[Title/Abstract] OR " old myocardial infraction "[Title/Abstract] OR " stable phase after acute coronary syndrome "[Title/Abstract] OR " stable myocardial infarction "[Title/Abstract] OR " stable phase of myocardial infarction "[Title/Abstract] OR " myocardial infarction "[Title/Abstract] OR " acute coronary syndrome "[Title/Abstract] OR " STEMI "[Title/Abstract] OR " NSTEMI "[Title/Abstract] OR " ST-elevated myocardial infarction "[Title/Abstract] OR " ST-elevation myocardial infarction "[Title/Abstract] OR " ST-elevated MI "[Title/Abstract] OR " STelevation MI "[Title/Abstract] OR " heart infarction "[Title/Abstract] OR " myocardial scar "[Title/Abstract] OR " myocardial scarring "[Title/Abstract] OR " scarred myocardium "[Title/Abstract] OR " infarct scar "[Title/Abstract] OR " infarct scars "[Title/Abstract] OR " infarct scarring "[Title/Abstract] OR " infarct heterogeneity "[Title/Abstract] OR " SCAD "[Title/Abstract]  #2 "Shexiang Baoxin Pill"[Title/Abstract] OR "Shexiang Baoxin"[Title/Abstract] OR " Shexiang Baoxin wan "[Title/Abstract]  OR " Heart-Protecting Musk Pill "[Title/Abstract] OR " Heart pill of musk "[Title/Abstract] OR " musk Moschus "[Title/Abstract]  #3 #1 AND #2  #4 ("randomized controlled trial"[Publication Type] OR "controlled clinical trial"[Publication Type] OR "randomized"[Title/Abstract] OR "placebo"[Title/Abstract] OR "clinical trials as topic"[MeSH Terms:noexp] OR "randomly"[Title/Abstract] OR "trial"[Title])  #5 #3 AND #4 |
| --- | --- |
| **Web of Science** | #5 #3 AND #4  #4 TS= (randomized controlled trial OR controlled clinical trial OR trial OR OR Clinical Trial OR random OR random allocation OR single-blind method OR double-blind method)  #3 #1 AND #2  #2 TS= (stable coronary artery disease OR stable coronary heart disease OR stable angina pectoris OR stable angina OR anginas OR stable coronary OR coronary artery disease OR ischemic cardiomyopathy OR ischaemic cardiomyopathy OR ischemic CM OR ischaemic CM OR ischemic heart disease OR ischaemic heart disease OR previous myocardial infarction OR old myocardial infraction OR stable phase after acute coronary syndrome OR stable myocardial infarction OR stable phase of myocardial infarction OR myocardial infarction OR acute coronary syndrome OR STEMI OR NSTEMI OR ST-elevated myocardial infarction OR ST-elevation myocardial infarction OR ST-elevated MI OR STelevation MI OR heart infarction OR myocardial scar OR myocardial scarring OR scarred myocardium OR infarct scar OR infarct scars OR infarct scarring OR infarct heterogeneity OR SCAD)  #1 TS= (Shexiang Baoxin Pill OR Shexiang Baoxin OR Shexiang Baoxin wan OR Heart-Protecting Musk Pill OR Heart pill of musk OR musk Moschus) |
| **Clinicaltrials.gov** | **Condition or disease:** (stable coronary artery disease OR stable coronary heart disease OR stable angina pectoris OR stable angina OR anginas OR stable coronary OR coronary artery disease OR ischemic cardiomyopathy OR ischaemic cardiomyopathy OR ischemic CM OR ischaemic CM OR ischemic heart disease OR ischaemic heart disease OR previous myocardial infarction OR old myocardial infraction OR stable phase after acute coronary syndrome OR stable myocardial infarction OR stable phase of myocardial infarction OR myocardial infarction OR acute coronary syndrome OR STEMI OR NSTEMI OR ST-elevated myocardial infarction OR ST-elevation myocardial infarction OR ST-elevated MI OR STelevation MI OR heart infarction OR myocardial scar OR myocardial scarring OR scarred myocardium OR infarct scar OR infarct scars OR infarct scarring OR infarct heterogeneity OR SCAD)  **Other terms:** (Shexiang Baoxin Pill OR Shexiang Baoxin OR Shexiang Baoxin wan OR Heart-Protecting Musk Pill OR Heart pill of musk OR musk Moschus) |
| **the Cochrane library** | Title Abstract Keyword=(Shexiang Baoxin Pill OR Shexiang Baoxin OR Shexiang Baoxin wan OR Heart-Protecting Musk Pill OR Heart pill of musk OR musk Moschus)  AND  Title Abstract Keyword= (stable coronary artery disease OR stable coronary heart disease OR stable angina pectoris OR stable angina OR anginas OR stable coronary OR coronary artery disease OR ischemic cardiomyopathy OR ischaemic cardiomyopathy OR ischemic CM OR ischaemic CM OR ischemic heart disease OR ischaemic heart disease OR previous myocardial infarction OR old myocardial infraction OR stable phase after acute coronary syndrome OR stable myocardial infarction OR stable phase of myocardial infarction OR myocardial infarction OR acute coronary syndrome OR STEMI OR NSTEMI OR ST-elevated myocardial infarction OR ST-elevation myocardial infarction OR ST-elevated MI OR STelevation MI OR heart infarction OR myocardial scar OR myocardial scarring OR scarred myocardium OR infarct scar OR infarct scars OR infarct scarring OR infarct heterogeneity OR SCAD)  AND  Title Abstract Keyword= (randomized controlled trial OR controlled clinical trial OR trial OR OR Clinical Trial OR random OR random allocation OR single-blind method OR double-blind method) |
| **CNKI** | ( SU='麝香保心丸' )  AND  (SU='稳定性冠心病' + '稳定型心绞痛' +'心绞痛' + '缺血性心肌病'+'缺血性心脏病'+ '急性冠脉综合征后稳定期' + '心肌梗死稳定期' + '陈旧性心肌梗死'+ '心肌梗死后' ) |
| **Wanfang** | 主题：(麝香保心丸)  AND  主题：(稳定性冠心病OR稳定型心绞痛OR心绞痛OR缺血性心肌病OR缺血性心脏病OR急性冠脉综合征后稳定期OR陈旧性心肌梗死 OR心肌梗死稳定期OR心肌梗死后) |
| **VIP** | (M=麝香保心丸)  AND  (M=稳定性冠心病OR稳定型心绞痛OR心绞痛OR缺血性心肌病OR缺血性心脏病OR急性冠脉综合征后稳定期OR陈旧性心肌梗死 OR心肌梗死稳定期OR心肌梗死后) |
| **SinoMed** | ("麝香保心丸"[常用字段:智能])  AND  ("稳定性冠心病"[常用字段:智能] OR"稳定型心绞痛"[常用字段:智能] OR"心绞痛"[常用字段:智能]OR"缺血性心肌病"[常用字段:智能] OR"缺血性心脏病"[常用字段:智能]OR "急性冠脉综合征后稳定期"[常用字段:智能]OR"陈旧性心肌梗死"[常用字段:智能] OR "心肌梗死后"[常用字段:智能] OR "心肌梗死稳定期"[常用字段:智能]) |

# Supplementary File S4. A list of excluded studies by reading the full text.

**Non-RCTs:**

[1]白瑶,许耀宗. 麝香保心丸治疗冠心病稳定性心绞痛的临床疗效及安全性研究[J]. 中药药理与临床,2015,31(1):308-309.

[2]王冬毅. 麝香保心丸联合西药治疗血瘀型稳定型心绞痛38例疗效分析[J]. 中医临床研究,2015(7):54-55. DOI:10.3969/j.issn.1674-7860.2015.7.028.

[3]杜学宏. 麝香保心丸治疗冠心病稳定性心绞痛患者的疗效[J]. 现代实用医学,2015,27(6):793-795. DOI:10.3969/j.issn.1671-0800.2015.06.054.

[4]庞文双. 麝香保心丸治疗冠心病稳定型心绞痛的疗效观察[J]. 中国社区医师,2007,23(10):43-44.

[5]胡帼英. 麝香保心丸治疗缺血性心脏病心绞痛的临床疗效观察[J]. 中成药,2008,30(2):312-附1. DOI:10.3969/j.issn.1001-1528.2008.02.063.

[6]赵季璇. 麝香保心丸治疗老年冠心病稳定性心绞痛60例临床观察[J]. 现代医院,2008,8(12):31-32. DOI:10.3969/j.issn.1671-332X.2008.12.015.

[7] 杜学宏. 麝香保心丸治疗冠心病稳定性心绞痛患者的疗效[J]. 现代实用医学,2015,27(6):793-795. DOI:10.3969/j.issn.1671-0800.2015.06.054.

[8] 都万卿.麝香保心丸和硝酸异山梨酯片联用治疗冠状动脉粥样硬化性心脏病心绞痛66例[J].中医临床研究,2017,9(16):24-26.

[9] 张旭光.麝香保心丸对老年稳定性心绞痛患者临床症状的影响[J].河南医学研究,2017,26(11):2013-2014.

[10] 王焕超,焦春艳,宓丽影,卢凤霞,马明祥.中西医联合治疗对冠心病心绞痛患者全血黏度、血浆黏稠度、纤维蛋白原含量的影响[J].临床合理用药杂志,2020,13(08):110-111.DOI:10.15887/j.cnki.13-1389/r.2020.08.064.

[11] 周艳鹏,马玉梅. 长期服用麝香保心丸治疗冠心病临床疗效观察[J]. 北方药学,2016,13(9):63-63.

[12] 陈剑飞. 中西医结合治疗老年稳定型心绞痛疗效观察[J]. 山西中医,2013,29(1):33-34. DOI:10.3969/j.issn.1000-7156.2013.01.018.

**Inconsistent interventions:**

[1]罗英. 35例老年稳定型心绞痛患者临床分析[J]. 中国保健营养（下旬刊）,2014,24(7):3748-3749. DOI:10.3969/j.issn.1004-7484(x).2014.07.074.

[2] 贾兴泽,赵婷丽,李运夏,等. 麝香保心丸联合地尔硫卓治疗冠心病心绞痛疗效观察[J]. 环球中医药,2014(s2):37-38.

**Inconsistent study purpose and outcome indicators:**

[1] 冯国杨. 冠心病稳定性心绞痛患者口服麝香保心丸的耐受性及安全性分析[J]. 中西医结合心血管病电子杂志,2016,4(31):155-156. DOI:10.3969/j.issn.2095-6681.2016.31.121.

[2] 杜伟. 冠心病稳定性心绞痛患者口服麝香保心丸的耐受性及安全性分析[J]. 临床医药文献电子杂志,2017,4(2):345,348. DOI:10.3877/j.issn.2095-8242.2017.02.116.

[3]彭雪梅,张武宁,余本凯,等. 麝香保心丸治疗老年慢性稳定性冠心病长期疗效的临床研究[J]. 中国保健营养（中旬刊）,2012(5):116-117.

[4] 罗应超,张金强,欧丽志. 166例麝香保心丸治疗稳定型心绞痛的临床疗效观察[J]. 医药前沿,2014(7):189-189. DOI:10.3969/j.issn.2095-1752.2014.07.199.

[5] Gao, J., Wang, X., Li, L., Zhang, H., He, R., Han, B., et al. (2021). Block Matching Pyramid Algorithm-Based Analysis on Efficacy of Shexiang Baoxin Pills Guided by Echocardiogram (ECG) on Patients with Angina Pectoris in Coronary Heart Disease. *J Healthc Eng.* 2021:3819900. doi:10.1155/2021/3819900.

**Duplicate publications:**

[1]贺金国,张蓓,王首军.麝香保心丸治疗缺血性心肌病的长期疗效观察[J].临床合理用药杂志,2013,6(30):15-16.DOI:10.15887/j.cnki.13-1389/r.2013.30.009.

[2]李公星. 麝香保心丸治疗稳定型心绞痛的临床观察[J]. 按摩与康复医学（上旬刊）,2012,3(7):72-74. DOI:10.3969/j.issn.1008-1879.2012.07.049.

[3]冉秀红. 麝香保心丸治疗冠心病稳定型心绞痛的疗效观察[J]. 中外医疗,2007,26(19):43-44. DOI:10.3969/j.issn.1674-0742.2007.19.037.

[4]胡嘉惠. 麝香保心丸治疗稳定型心绞痛的临床疗效观察[J]. 中国中医药咨讯,2010,2(32):37-38.

# Supplementary File S5. The follow-up times for all outcome measures.

| Outcome | Follow-up times | |
| --- | --- | --- |
|  | ≤ 6 months | > 6 months |
| 1.MACE | Zhao and Xie, 2021, Gao and Chen, 2021, Ding, 2016, Yang et al., 2013 | Ge et al., 2020, Peng et al., 2017 |
| 2.The total effective rate of angina symptom improvement | Zhao et al., 2022, Pan et al., 2022, Xia et al., 2021, Gao and Chen, 2021, Wang ang Zhu, 2020, Chen and Chen, 2020, Zhao et al., 2018, Zhao, 2018, Wang et al., 2018, Liu, 2018, Xia, 2016, Ding, 2016, Wang et al., 2015, Tan, 2015, Ji, 2015, Hou, 2015, Wang, 2014, Liu, 2014, Huang et al., 2014, Chen, 2014, Sun, 2013, Lv, 2012, Guo and Tan, 2012, Shi and Hang, 2011, Xu et al., 2010, Sun, 2010 | Wang, 2016 |
| 3.ECG improvement | Pan et al., 2022, Gao and Chen, 2021, Wang ang Zhu, 2020, Zhao et al., 2018, Wang et al., 2018, Xia, 2016, Ji, 2015, Hou, 2015, Zhao, 2014, Wang, 2014, Liu, 2014, Sun, 2013, Lv, 2012, Guo and Tan, 2012, Xu et al., 2010, Sun, 2010, Huang et al., 2014 | no study |
| 4.AEs | Zhao and Xie, 2021, Gao and Chen, 2021, Wang ang Zhu, 2020, Liu, 2018, Xia, 2016, Ji, 2015, Hou, 2015, Zhao, 2014, Wang, 2014, Chen, 2014, Zou et al., 2013, Sun, 2013, Lv, 2012, Guo and Tan, 2012, Xu et al., 2010, Pan, 2016 | Ge et al., 2020, Peng et al., 2017 |
| 5.Angina pectoris frequency | Yang, 2022, Zhao et al., 2022, Pan et al., 2022, Zhou, 2021, Xie and Huang, 2021, Xia et al., 2021, Chen and Chen, 2020, Wang et al., 2015, Tan, 2015, Ji, 2015, Guo and Tan, 2012, Xu et al., 2010 | Peng et al., 2017 |
| 6.Angina pectoris duration | Yang, 2022, Zhao et al., 2022, Zhou, 2021, Xie and Huang, 2021, Xia et al., 2021, Chen and Chen, 2020, Ji, 2015, Guo and Tan, 2012 | no study |
| 7.LVEF | Yang, 2022, Xu et al., 2010, Wang, 2018, Miao et al., 2016, Liao and Huang, 2019, Pan, 2016, Wu et al., 2012, Jiang, 2012 | no study |
| 8.Blood lipid index | Zhao et al., 2022, Pan et al., 2022, Xia et al., 2021, Zhao, 2014, Lv, 2012, Xu et al., 2010, Miao et al., 2016 | Wang, 2016 |

#

# Supplementary File S6. ROB 2.0 for outcomes.

**6.1 ROB for MACE**

**6.2 ROB for the total effective rate of angina symptom improvement**

**6.3 ROB for ECG improvement**

**6.4 ROB for AEs**

**6.5 ROB for angina pectoris frequency**

**6.6 ROB for angina pectoris duration**

**6.7 ROB for LVEF**

**6.8 ROB for blood lipid level**

# Supplementary File S7. Adverse drug reactions or adverse events.

| Studies | Adverse drug reactions or adverse events | |
| --- | --- | --- |
|  | the experimental group | the control group |
| Ge, 2021 | 236 cases of AEs（hepatobiliary disease, renal and urinary system diseases, and metabolic and nutritional diseases） | 231 cases of AEs（hepatobiliary disease, renal and urinary system diseases, and metabolic and nutritional diseases） |
| Zhao and Xie, 2021; | 2 cases of gastrointestinal intolerance | 6 cases of gastrointestinal intolerance |
| Gao and Chen, 2021 | 5 cases of AEs (specific unknown) | 6 cases of AEs (specific unknown) |
| Wang and Zhu, 2020 | 2 cases of gastrointestinal intolerance, 2 cases of dizziness | 1 case of gastrointestinal intolerance |
| Liu, 2018 | 0 | 1 case of gastrointestinal intolerance, 2 cases of dizziness |
| Peng et al., 2017 | 2 cases of gastrointestinal intolerance | 0 |
| Xia, 2016 | 5 cases of headache | 16 cases of headache, 2 cases of hypotension |
| Ji, 2015 | 1 case of angina aggravation | 1 case of angina aggravation |
| Hou，2015 | 3 cases of headache, 1 case of gastrointestinal intolerance | 2 cases of headache |
| Zhao, 2014 | 0 | 8 cases of headache |
| Wang, 2014 | 1 case of tongue numbness | 2 cases of headache, 1 case of gastrointestinal intolerance |
| Chen, 2014 | 1 case of dizziness | 2 cases of dizziness, 2 cases of gastrointestinal intolerance, and 1 case of rash |
| Zou et al., 2013 | 3 cases of headache | 2 cases of gastrointestinal intolerance |
| Sun, 2013 | 4 cases of tongue numbness | 0 |
| Lv, 2012 | 13 cases of gastrointestinal intolerance | 0 |
| Guo and Tan, 2012 | 2 cases of gastrointestinal intolerance, 2 cases of tongue numbness | 5 cases of headache |
| Xu et al., 2010 | 1 case of gastrointestinal intolerance, 1 case of rash | 0 |
| Pan, 2016 | 2 cases of gastrointestinal intolerance | 11 cases of gastrointestinal intolerance |

# Supplementary File S8. Results of meta-regression analysis.

| Outcome or variable | *t* | *P* | 95% CI |
| --- | --- | --- | --- |
| 1.Angina Pectoris Frequency |  |  |  |
| Publication year | 0.02 | 0.99 | (-0.81, 0.82) |
| Mean age (<55 / <65 and ≥ 55 / ≥ 65) | 1.08 | 0.30 | (-3.25, 9.48) |
| Treatment duration | -1.23 | 0.24 | (-1.70, 0.48) |
| Sample size (≥ 100 / < 65) | 1.50 | 0.16 | (-1.93, 10.24) |
| 2.Angina Pectoris Duration |  |  |  |
| Publication year | 0.28 | 0.79 | (-0.18, 0.22) |
| Mean age (<55 / <65 and ≥ 55 / ≥ 65) | -0.97 | 0.37 | (-1.78, 0.77) |
| Treatment duration | -6.85 | 0.00 | (-0.3, -0.14) |
| Sample size (≥ 100 / < 65) | 1.54 | 0.17 | (-0.45, 1.97) |
| 3.LVEF |  |  |  |
| Publication year | 1.88 | 0.11 | (-0.13, 0.95) |
| Mean age (<55 / <65 and ≥ 55 / ≥ 65) | -1.27 | 0.25 | (-4.04, 1.27) |
| Treatment duration | -0.95 | 0.38 | (-3.62, 1.59) |
| Sample size (≥ 100 / < 65) | -0.98 | 0.37 | (-9.54, 4.10) |
| 4.TC |  |  |  |
| Publication year | -0.53 | 0.62 | (-0.08, 0.05) |
| Mean age (<55 / <65 and ≥ 55 / ≥ 65) | 1.21 | 0.28 | (-0.27, 0.76) |
| Treatment duration | -0.76 | 0.48 | (-0.28,0.15) |
| Sample size (≥ 100 / < 65) | 0.71 | 0.51 | (-0.42, 0.74) |
| 5.TG |  |  |  |
| Publication year | -1.85 | 0.12 | (-0.08, 0.01) |
| Mean age (<55 / <65 and ≥ 55 / ≥ 65) | 1.98 | 0.09 | (-0.08, 0.77) |
| Treatment duration | 0.04 | 0.97 | (-0.15, 0.15) |
| Sample size (≥ 100 / < 65) | 2.68 | 0.04 | (0.03, 0.74) |
| 6.LDL-C |  |  |  |
| Publication year | -2.02 | 0.09 | (-0.03, 0.00) |
| Mean age (<55 / <65 and ≥ 55 / ≥ 65) | -0.31 | 0.77 | (-0.26, 0.20) |
| Treatment duration | 0.94 | 0.39 | (-0.03, 0.078) |
| Sample size (≥ 100 / < 65) | 0.86 | 0.42 | (-0.13, 0.28) |
| 7.HDL-C |  |  |  |
| Publication year | 1.25 | 0.33 | (-0.06, 0.11) |
| Mean age (<55 / <65 and ≥ 55 / ≥ 65) | -2.54 | 0.13 | (-0.96, 0.25) |
| Treatment duration | -0.70 | 0.56 | (-0.43, 0.31) |
| Sample size (≥ 100 / < 65) | -1.24 | 0.34 | (-1.38, 0.76) |

# Supplementary File S9. Results of subgroup analysis

## 9.1 Subgroup analysis of AEs according to the treatment duration.


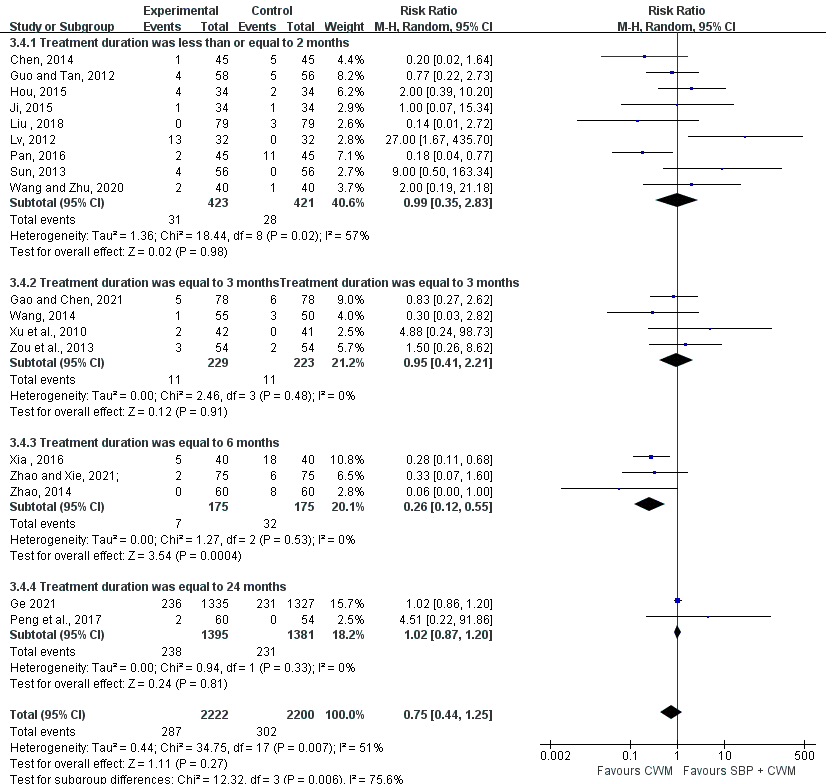


## 9.2 Subgroup analysis of angina pectoris frequency according to the treatment duration.


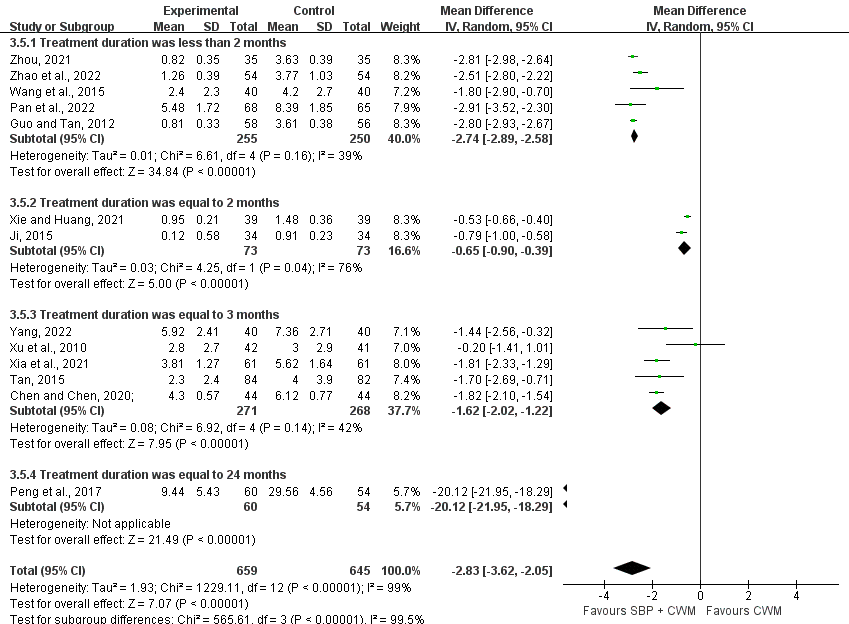


## 9.3 Subgroup analysis of angina pectoris duration according to the treatment duration.
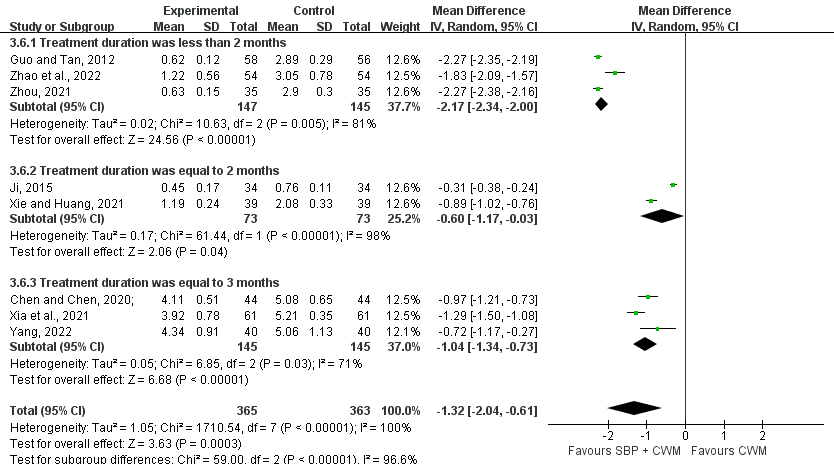


## 9.4 Subgroup analysis of LVEF according to the treatment duration.


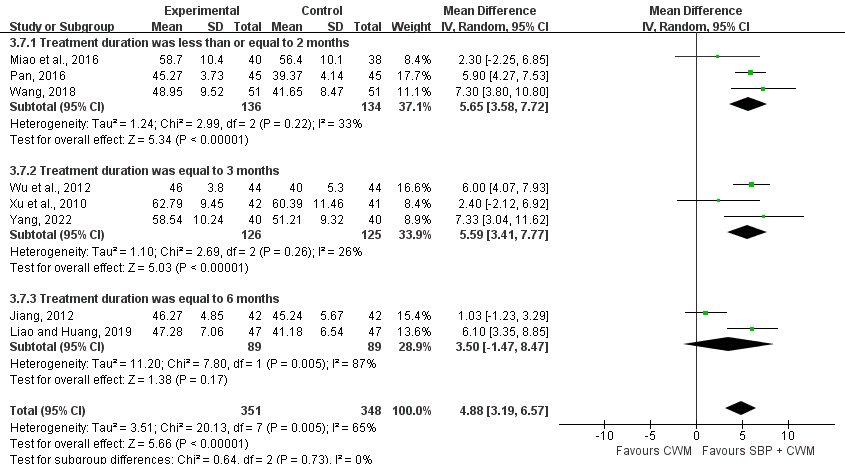


## 9.5 Subgroup analysis of TC according to the treatment duration.


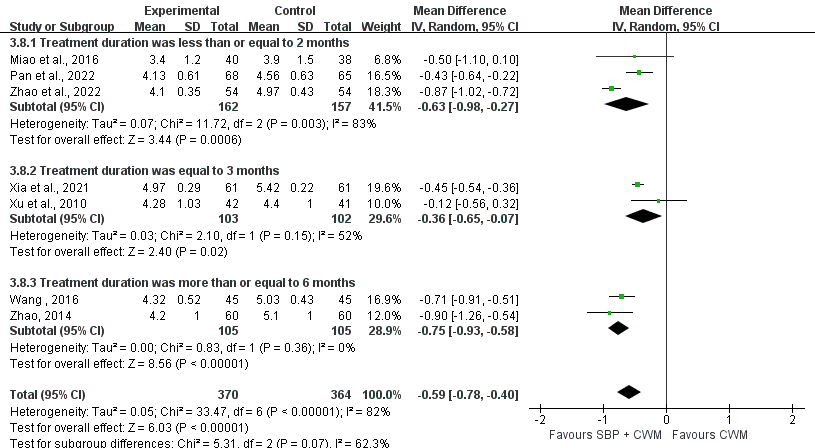


## 9.6 Subgroup analysis of TG according to the treatment duration

##
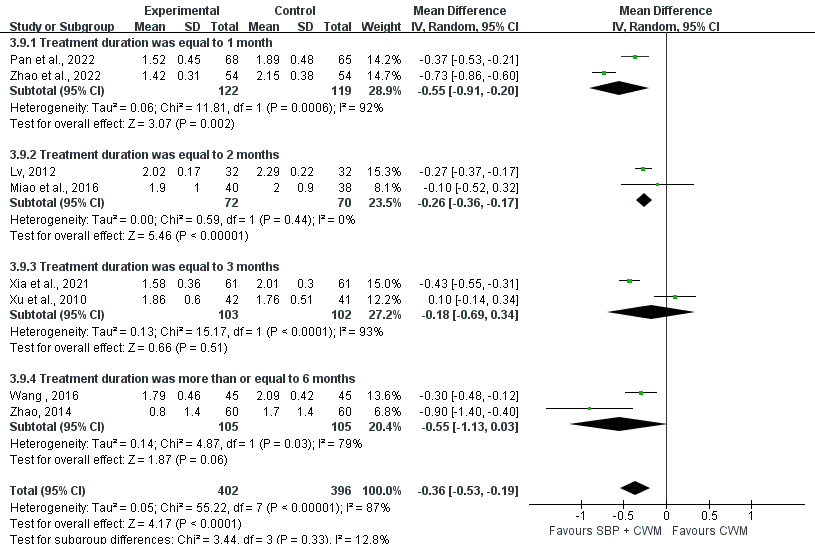


## 9.7 Subgroup analysis of LDL-C according to the treatment duration.


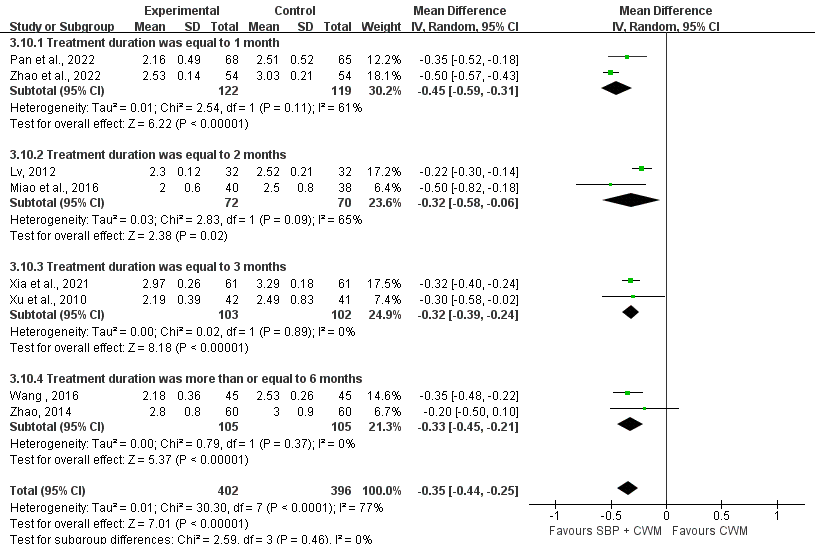


## 9.8 Subgroup analysis of HDL-C according to the treatment duration.


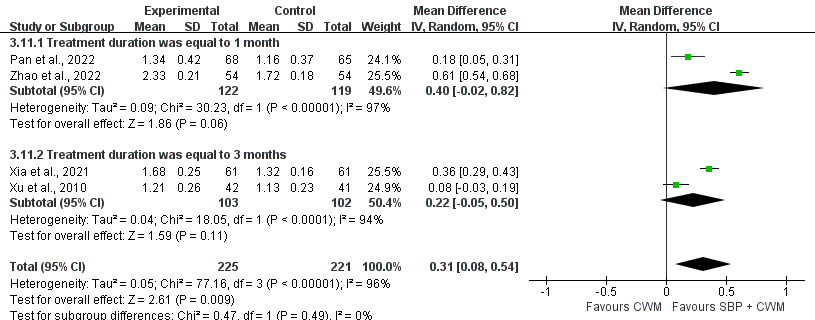


| Supplementary File S10. Results of sensitivity analysis |
| --- |

## 10.1 Sensitivity analysis of MACE.


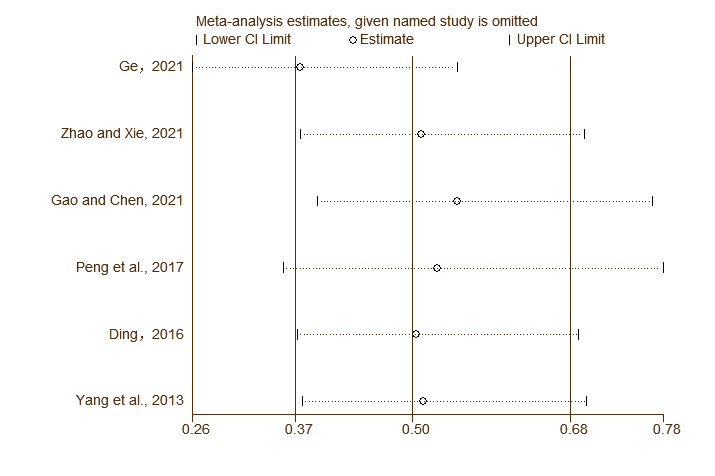


## 10.2 Sensitivity analysis of the total effective rate of angina symptom improvement.


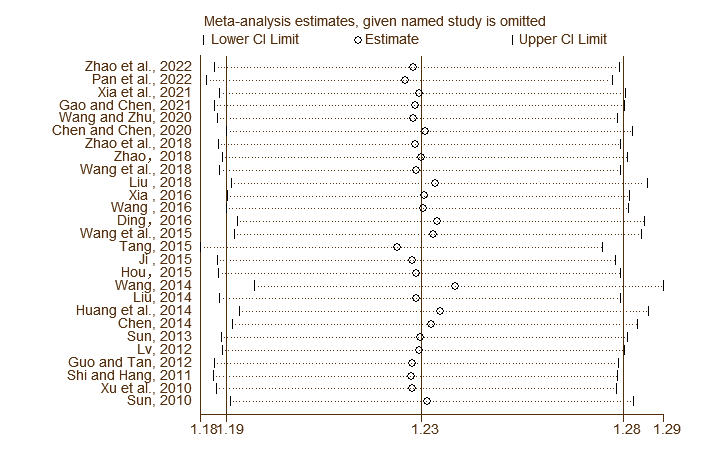


## 10.3 Sensitivity analysis of ECG Improvement.


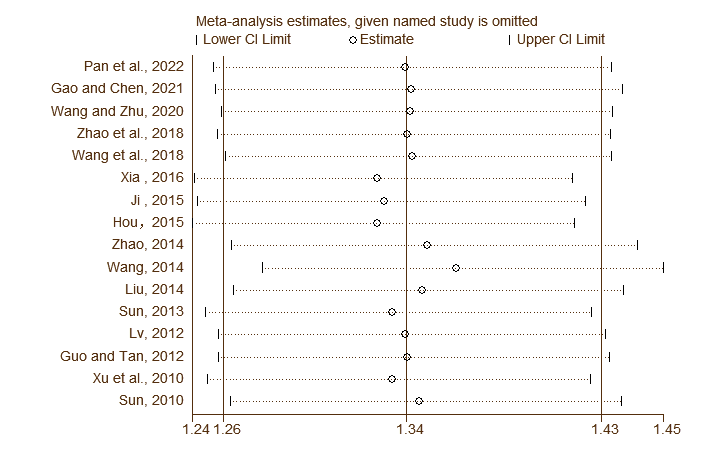


## 10.4 Sensitivity analysis of AEs.


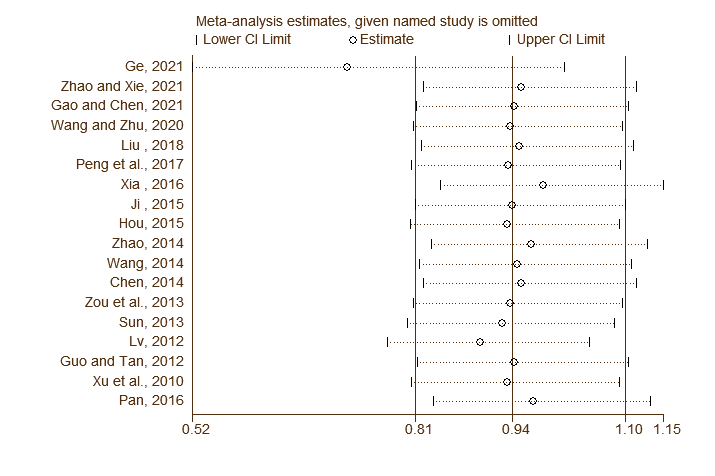


# Supplementary File S11. Egger’s test of the outcomes.

## 11.1 Egger’s test of the total effective rate of angina symptom improvement.


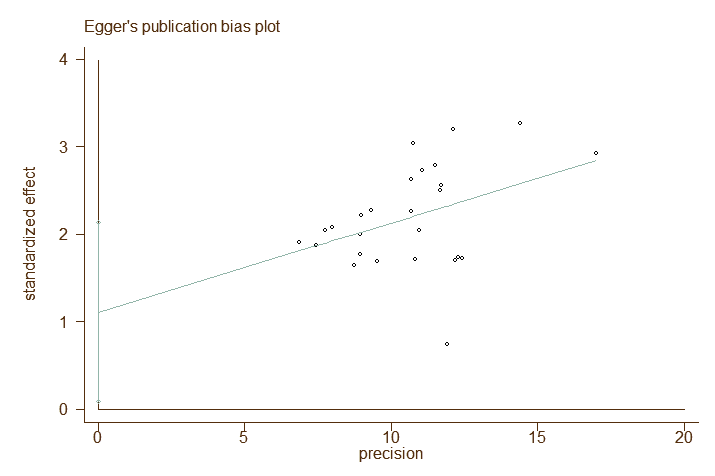


## 11.2 Egger’s test of ECG improvement.


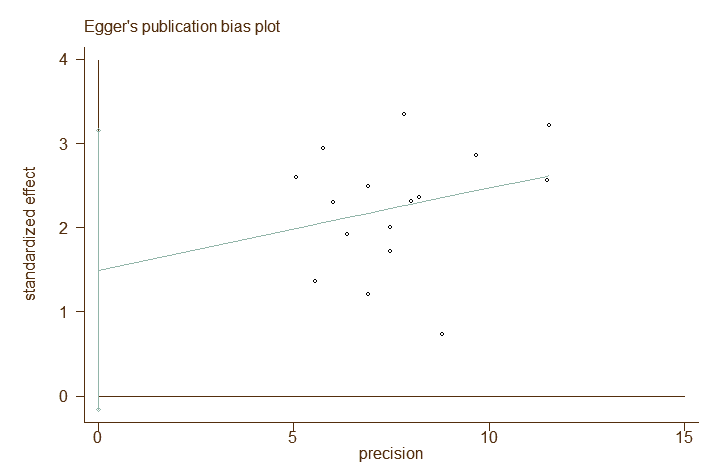


## 11.3 Egger’s test of AEs.


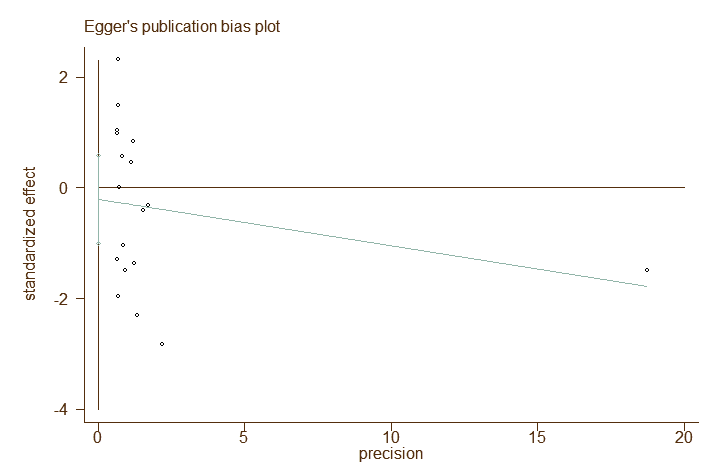


## 11.4 Egger’s test of angina pectoris frequency.


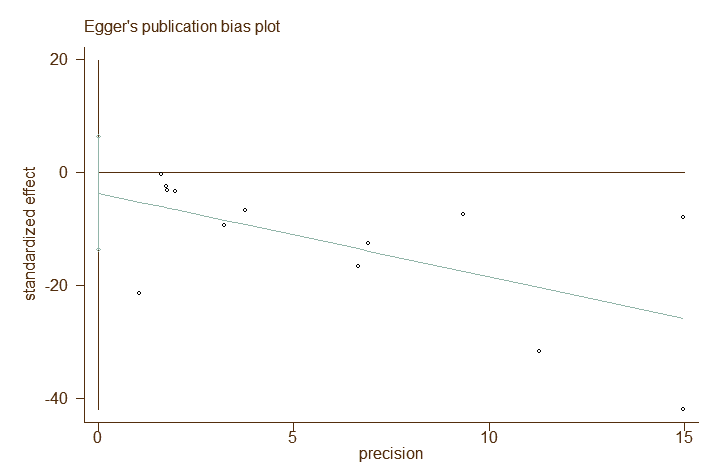

Supplement: Supplementary file 1 [file DataSheet1.docx]
